# Intimate relationships among actinomycetes and mycolic acid-containing bacteria

**DOI:** 10.1038/s41598-022-11406-2

**Published:** 2022-05-04

**Authors:** Manami Kato, Shumpei Asamizu, Hiroyasu Onaka

**Affiliations:** 1 Graduate School of Agricultural and Life Sciences, The University of Tokyo, 1-1-1 Yayoi, Bunkyo, Tokyo, 113-8657 Japan; 2 Collaborative Research Institute for Innovative Microbiology, The University of Tokyo, 1-1-1 Yayoi, Bunkyo, Tokyo, 113-8657 Japan

**Keywords:** Antibiotics, Microbial ecology, Soil microbiology

## Abstract

Co-culture is an efficient strategy for natural product discovery. We have used mycolic acid-containing bacteria (MACB) *Tsukamurella pumonis* TP-B0596 to induce secondary metabolism by actinomycetes and have found several natural products. We also observed that MACB attached to the mycelium of *Streptomyces lividans* forming coaggregates during combined-culture. This stimulated interest in the interactions among actinomycetes and MACB, and we found that soil isolated cultures contained a mixture of actinomycetes and MACB. Our previously observed interactions were the result of selective screening and combination of bacteria in the lab, which warranted investigation of the existence of these interactions in the natural soil environment. Therefore, in this paper, we report the interaction between a co-isolated natural pair of actinomycetes and MACB in terms of morphology and metabolic changes. A natural pair of actinomycetes and MACB co-aggregated in liquid culture and showed metabolic changes. Interestingly, co-aggregated actinomycetes and MACB were re-isolated from soil with no obvious morphological colony differences from the colony of a single strain. The results demonstrate that there is a stochastic chance of picking colonies containing co-aggregated actinomycetes and MACB, which suggests that the pair can exist in co-aggregate form in the soil environment and interact with each other.

## Introduction

Actinomycetes are Gram-positive soil bacteria that produce pharmaceutically important natural products as secondary metabolites^1^. Each strain contains more than 30 putative biosynthetic gene clusters for secondary metabolites^2,3^. Although they are considered to have potential for producing more than 30 secondary metabolites, the number of detectable products in normal lab culture conditions are very limited^3,4^. Previous reports have shown that responses to environmental factors affect secondary metabolism of actinomycetes^4^. As many of bacterial interaction contains uninterpreted mechanism involving alteration of environmental factors, and thereby excite the response systems intricately, application of bacterial interaction will be a promising strategy for the activation of the cryptic biosynthetic genes for secondary metabolites, which were demonstrated by a number of reports.

The soil environment from where actinomycetes can be isolated contains many different microorganisms^5^. Actinomycetes respond to environmental factors including biogenic factors of other microorganisms^4^. We previously reported that a group of mycolic acid-containing bacteria (MACB; e.g. *Tsukamurella pulmonis* TP-B0596) affected the secondary metabolism of actinomycetes in lab culture^6^. MACB are actinomycetes containing specific long chain fatty acids (mycolic acids) on the cell wall^7,8^. Using MACB (such as *T. pulmonis* TP-B0596) as a partner, *Streptomyces lividans* TK23 was activated to produce undecylprodigiosin (RED) and actinorhodin (ACT), which are not produced under general lab culture conditions^6^. Using *T. pulmonis* TP-B0596 as a combined-culture partner, a number of natural products have been isolated^9–17^, and this strategy was also found to be efficient to enhance production during heterologous expression^18–20^. This co-culture method using the combination of actinomycetes and MACB is named combined-culture (see Supplemental Table 1 for the whole list).

The activation of RED and ACT did not occur through provision of culture extracts or killed bacteria^21^, therefore, it was suggested that physical cell contact by living *T. pulmonis* is required for the activation of RED and ACT by *S. lividans*. Our previous analysis of cell morphology of *S. lividans* and several MACB in liquid culture under scanning electron microscopy (SEM) imaging showed that they formed co-aggregates^21^. *Streptomyces* species generally grow by forming a pellet in liquid culture^22^, and significant attachment of MACB cells on *Streptomyces* mycelium was observed^21^. Co-aggregation and the production of RED and ACT was not observed with addition of killed MACB (e.g. by γ-ray irradiation or formaldehyde fixation)^21^. This suggested that co-aggregates formation through continuous physical contact might be important for the activation of secondary metabolism by actinomycetes.

Bacteria can form interspecies aggregates in the environment^23^, and these aggregates can also occur between bacteria from different environments^24^. Several theories have been reported to explain the interactions in interspecies aggregates for oral or sludge environments^23^. However, there is limited understanding of the mechanism and function of *Streptomyces*/MACB aggregates in the general soil bacterial community^25^, and it will be important to elucidate the ecological relevance or benefits of those bacterial interactions in the future.

The previous observation of the interactions between actinomycetes and MACB was the result of deliberate combinations of bacteria selected by screening in the lab for activation of secondary metabolism^6,21^. However, these results suggested that the characteristic interactions such as forming interspecies co-aggregates and affecting each other including secondary metabolism, might be naturally occurring events in the soil environment. In this study, we report the morphology and metabolic changes of a co-isolated pair of actinomycetes and MACB from environmental soil of Hegura Island. The natural co-isolated pair formed interspecies aggregates and their interaction under combined-culture lab conditions affected the production of secondary metabolites. We performed additional experiments to re-isolate the co-aggregated cell pellets and demonstrated that mixed aggregates of actinomycetes and MACB were co-isolated with no obvious morphological change of the growing colony.

## Results

### Isolation of actinomycetes from Hegura Island

**Figure 1:**
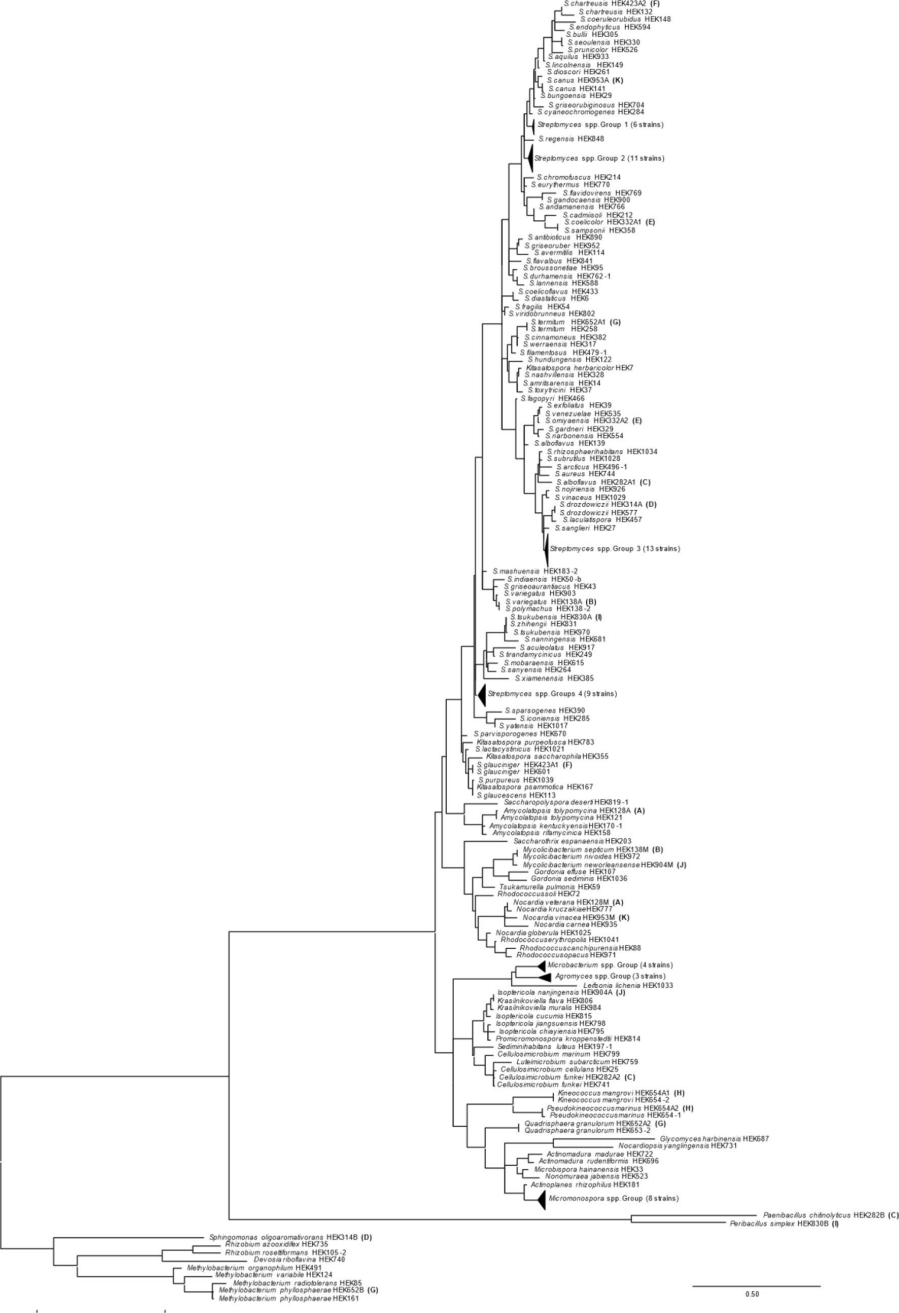
Phylogenetic tree of isolated bacteria. Parenthesized numbers indicate the number of strains within the clade. Accession numbers are of the most similar type strains. Letters A–K indicate the co-isolated bacterial pairs.

Hegura Island (N37°51′5″, E136°55′7″) in Ishikawa prefecture, Japan, is known as a stopover point for migratory wild birds in the middle of the Sea of Japan. We expected that those migratory wild birds might bring bacteria associated to their surface and stool and give rich bacterial diversity to this isolated island. We collected environmental samples from 71 points on Hegura Island in June 2008. General methods were applied for the isolation of actinomycetes (see “Materials and methods” for detail and Supplemental Fig. 1); growing single colonies that appeared to be actinomycetes were isolated from the agar plates. We obtained a total of 1041 bacterial isolates from this process. Partial 16S rRNA gene sequences of randomly selected 182 isolates were determined to estimate the taxonomic diversity of the obtained in-house culture collection. The culture collection contained 182 different bacterial species, including *Streptomyces* species (63.7%, 116/182), non-*Streptomyces* actinomycetes (26.4%, 48/182), MACB (6.0%, 11/182), and other bacteria (3.8%, 7/182). (Fig. 1, Supplemental Tables 2 and 3).

### Identification of co-isolated strains

After making the spore/cell stocks of the isolated strains, stock solutions were inoculated onto ISP2 agar plates, which was different from the original isolation medium (Supplemental Fig. 1). On those agar plates, we observed at least 11 stocks that appeared to contain more than two different bacteria (Table 1). Although the isolates were thoroughly separated in the isolation process including vigorous agitation by vortex mixer and considerable dilution, we obtained mixed colonies of more than two bacteria. Therefore, it was speculated that those bacteria originally formed tight co-aggregates in soil and thus were isolated as a single strain but were subsequently found to be a mixture of several strains. We defined those bacteria as co-isolated bacteria and performed further analysis.

**Table 1:** Identification of strains from co-isolated colonies.

Strain No	Identified strain		Most similar type strain	Matched/total nt	Identity (%)	Order	Accession No
HEK128	*Amycolatopsis tolypomycina* HEK128A	A	*A. tolypomycina* JCM 4936	1465/1482	98.9	*Pseudonocardiales*	AJ508241
	*Nocardia veterana* HEK128M	MACB	*N. veterana* JCM 11307	1457/1460	99.8	*Corynebacteriales*	JN041602
HEK138	*Streptomyces variegatus* HEK138A	A	*S. variegatus* JCM 6930	1433/1451	98.8	*Streptomycetales*	AB184688
	*Mycolicibacterium septicum* HEK138M	MACB	*M. septicum* JCM 14743	1475/1475	100.0	*Corynebacteriales*	AF547964
HEK282	*Streptomyces alboflavus* HEK282A1	A	*S. alboflavus* JCM 4615	1336/1357	98.5	*Streptomycetales*	EF178699
	*Cellulosimicrobium funkei* HEK282A2	A	*C. funkei* JCM 14302	1362/1363	99.9	*Micrococcales*	AY501364
	*Paenibacillus chitinolyticus* HEK282B	GP	*P. chitinolyticus* JCM 12162	1385/1398	99.1	*Bacillales*	AB680938
HEK314	*Streptomyces* sp. HEK314A	A	*S. drozdowiczii* JCM 13580	1357/1361	97.8	*Streptomycetales*	AF529199
	*Sphingomonas oligoaromativorans* HEK314B	GN	*S. oligoaromativorans* NBRC105508	1319/1323	99.7	*Proteobacteria*	FJ434127
HEK332	*Streptomyces coelicolor* HEK332A1	A	*S. coelicolor* JCM4357	1361/1361	100.0	*Streptomycetales*	GU383131
	*Streptomyces omiyaensis* HEK332A2	A	*S. omiyaensis* JCM 4806	1359/1360	99.9	*Streptomycetales*	MT760618
HEK423	*Streptomyces glauciniger* HEK423A1	A	*S. glauciniger* JCM 12278	1343/1344	99.9	*Streptomycetales*	AB249964
	*Streptomyces chartreusis* HEK423A2	A	*S. chartreusis* JCM 4570	1385/1398	99.1	*Streptomycetales*	MT760575
HEK652	*Streptomyces termitum* HEK652A1	A	*S. termitum* JCM 4518	1358/1358	100.0	*Streptomycetales*	AB122742
	*Quadrisphaera granulorum* HEK652A2	A	*Q. granulorum* JCM 16010	1342/1358	98.8	*Kineosporiales*	AY831385
	*Methylobacterium phyllosphaerae* HEK652B	GN	*M. phyllosphaerae* JCM 16408	1315/1315	100.0	*Hyphomicrobiales*	EF126746
HEK654	*Kineococcus mangrovi* HEK654A1	A	*K. mangrovi* NBRC 110933	1354/1361	99.5	*Kineosporiales*	LC056925
	*Pseudokineococcus marinus* HEK654A2	A	*P. marinus* NBRC 102111	1333/1341	99.4	*Kineosporiales*	DQ200982
HEK830	*Streptomyces tsukebensis* HEK830A	A	*S. tsukubensis* NBRC 108819	1338/1349	99.2	*Streptomycetales*	AB217600
	*Peribacillus simplex* HEK830B	GP	*P. simplex* NBRC 15720	1387/1390	99.8	*Bacillales*	X60638
HEK904	*Isoptericola nanjingensis* HEK904A	A	*I. nanjingensis* DSM 24300	1352/1359	99.5	*Micrococcales*	HQ222356
	*Mycolicibacterium neworleansense* HEK904M	MACB	*M. neworleansense* JCM15659	1346/1347	99.9	*Corynebacteriales*	AY012575
HEK953	*Streptomyces canus* HEK953A	A	*S. canus* NBRC 12752	1350/1352	99.9	*Streptomycetales*	AB184118
	*Nocardia vinacea* HEK953M	MACB	*N. vinacea* JCM10988	1351/1356	99.6	*Corynebacteriales*	JN041530

*FA*, filamentous actinomycetes; *MACB*, mycolic acid-containing bacteria; *CA*, cocci-shaped actinomycetes; *GP*, Gram-positive bacteria; *GN*, Gram-negative bacteria; *A*, actinomycetes. Accession numbers are of the most similar type strains.

To identify the strains in the mixture, the dilution method was applied to obtain single colonies from the 11 mixed stocks. This procedure successfully isolated two to three different colony types growing on the agar plate with different colony morphology. We then grew the bacteria in liquid culture to isolate their genomic DNA and determined partial 16S rRNA gene sequences of each strain obtained from the 11 co-isolated colonies. Among these pairs, we identified four that were a mixture of actinomycetes and MACB, and seven that were a mixture of actinomycetes and other bacteria (Table 1).

### Co-aggregation of co-isolated actinomycetes and MACB

*S. lividans* TK23 (Sl) and *T. pulmonis* TP-B0596 (Tp) formed co-aggregates, where cells of Tp were attached to pellets of Sl. Therefore, it was predicted that the naturally co-isolated pairs of actinomycetes and MACB would have an affinity for each other and thus form similar mixed aggregates. To test this prediction, we grew the naturally co-isolated pair of actinomycetes and MACB in combined-culture and observed the morphological interaction by SEM imaging. The pairs of actinomycetes and MACB (HEK128A/M, HEK138A/M, HEK904A/M, HEK953A/M) were cultured in liquid medium, and the cultured cells were fixed for SEM imaging. From now on, we will use the letters A for actinomycetes, M for MACB, and B for other bacteria as abbreviation for strain identification.

**Figure 2:**
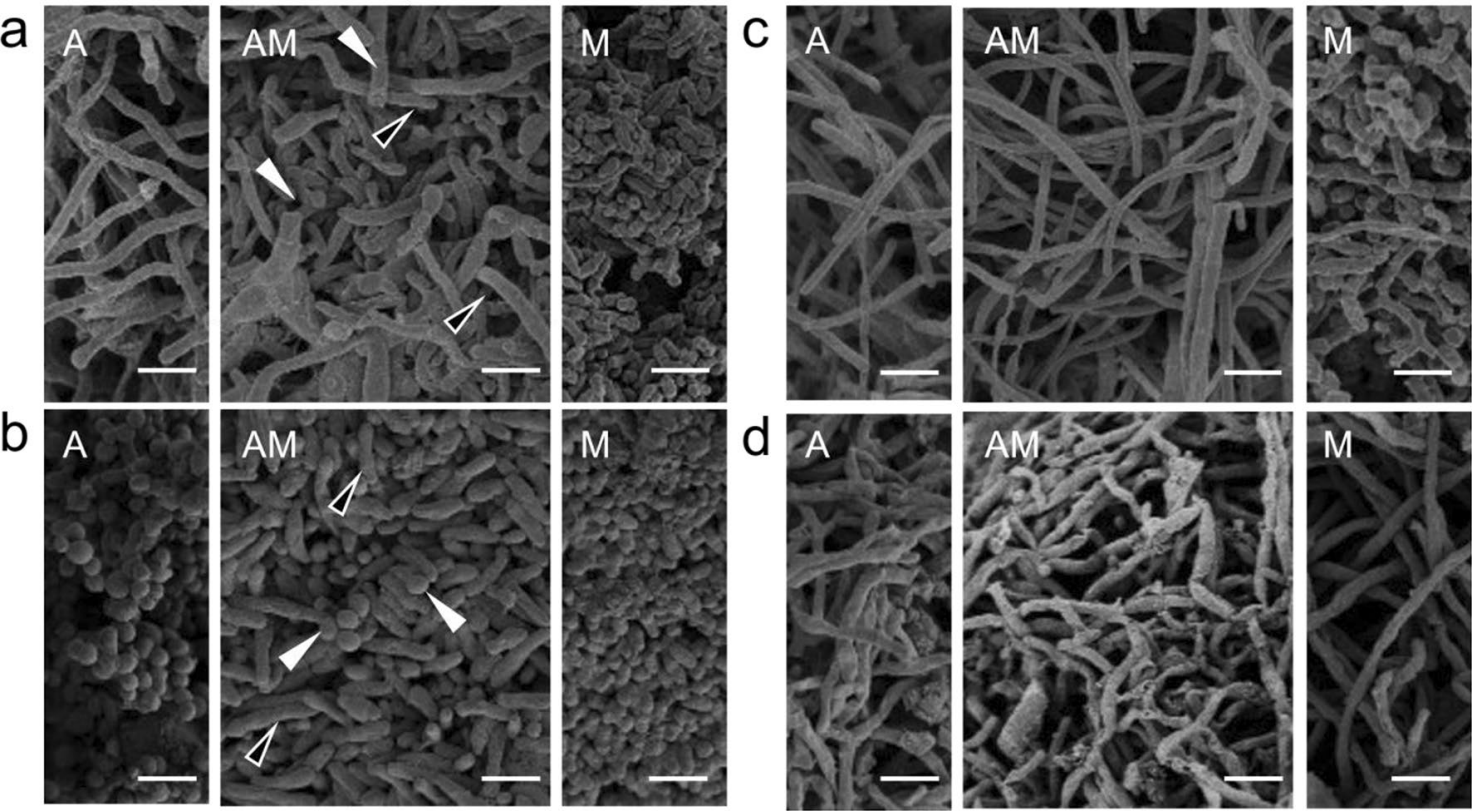
Scanning electron microscope image of co-isolated filamentous or cocci-shaped actinomycetes and mycolic acid-containing bacteria in combined-culture. (**a**) Left image: monoculture of HEK138A. Middle image: combined-culture of HEK138A/M. Right image: monoculture of HEK138M. White arrow indicates HEK138A filamentous cells and blank arrow indicates HEK138M bacilli-shaped cell. (**b**) Left image: monoculture of HEK904A. Middle image: combined-culture of HEK904A/M. Right image: monoculture of HEK904M. White arrow indicates HEK904A cocci-shaped cell and blank arrow indicates HEK904M cocci-shaped cell. (**c**) Left image: monoculture of HEK128A. Middle image: combined-culture of HEK128A/128 M. Right image: monoculture of HEK128M. (**d**) Left image: monoculture of HEK953A. Middle image: combined-culture of HEK953A/M. Right lower image: monoculture of HEK953M. (Scale bar = 2 µm).

HEK138, a co-isolated pair from bamboo rhizospheric soil using H_2_O dilution and ISP4 agar plate (Supplemental Table 4), contained the filamentous actinomycete *Streptomyces variegatus* HEK138A^26^ and MACB *Mycolicibacteirum septicum* HEK138M^27^ (Table 1). As expected, cells of *M. septicum* HEK138M (bacilli-shaped cells) and *S. variegatus* HEK138A (filamentous hyphae) generated co-aggregates, where the cell clump of *M. septicum* HEK138M coexisted with the cell pellet of *S. variegatus* HEK138A mycelium (Fig. 2a).

HEK904, a co-isolated pair from soil using phenol treatment and ISP4 agar plate (Supplemental Table 4), contained the cocci-shaped actinomycete *Isoptericola nanjingensis* HEK904A^28^ and MACB *Mycolicibacterium neworleansense* HEK904M^29^ (Table 1). The cocci-shaped actinomycete *I. nanjingensis* HEK904A and MACB *M. neworleansense* HEK904M also formed mixed aggregates. Although *I. nanjingensis* did not have the filamentous cell shape, *M. neworleansense* cells were bacilli-shaped, and the two strains could be differentiated by cell size (Fig. 2b). Therefore, SEM imaging confirmed that these two strains formed a mixed aggregate.

HEK128, a co-isolated pair from an unknown succulent plant rhizosphere soil using H_2_O dilution and humic acid agar plate (Supplemental Table 4), contained the filamentous actinomycete *Amycolatopsis tolypomycina* HEK128A^30^ and filamentous MACB *Nocardia veterana* HEK128M^31^ (Table 1). HEK953, a co-isolated pair from seaweed using H_2_O dilution and humic acid agar plate (Supplemental Table 4), contained the filamentous actinomycete *Streptomyces canus* HEK953A^32^ and filamentous MACB *Nocardia vinacea* HEK953M (Table 1). For these combinations (HEK128 and HEK953), because both strains had a filamentous cell shape, it was not possible to discriminate between them by SEM imaging (Fig. 2c and d). Therefore, we could not conclude whether these pairs formed mixed aggregates.

### Co-aggregation of co-isolated actinomycetes and other bacteria

Seven other co-isolated colonies were identified as mixtures of actinomycetes and other bacteria (Table 1). Four combinations were obtained from the remaining seven co-isolated stocks: *Streptomyces*–other bacteria (HEK314 and HEK830), cocci-shaped actinomycetes–cocci-shaped actinomycetes (HEK654), *Streptomyces*–cocci-shaped actinomycetes–other bacteria (HEK652 and HEK282), and *Streptomyces*–*Streptomyces* (HEK332 and HEK423) (Table 1). All the co-isolated bacteria could grow in monoculture, indicating that there was no obligatory symbiotic relationship between the co-isolates. As naturally co-isolated actinomycetes and MACB pairs formed coaggregates, we also tested these combinations for their ability to form coaggregates.

**Figure 3:**
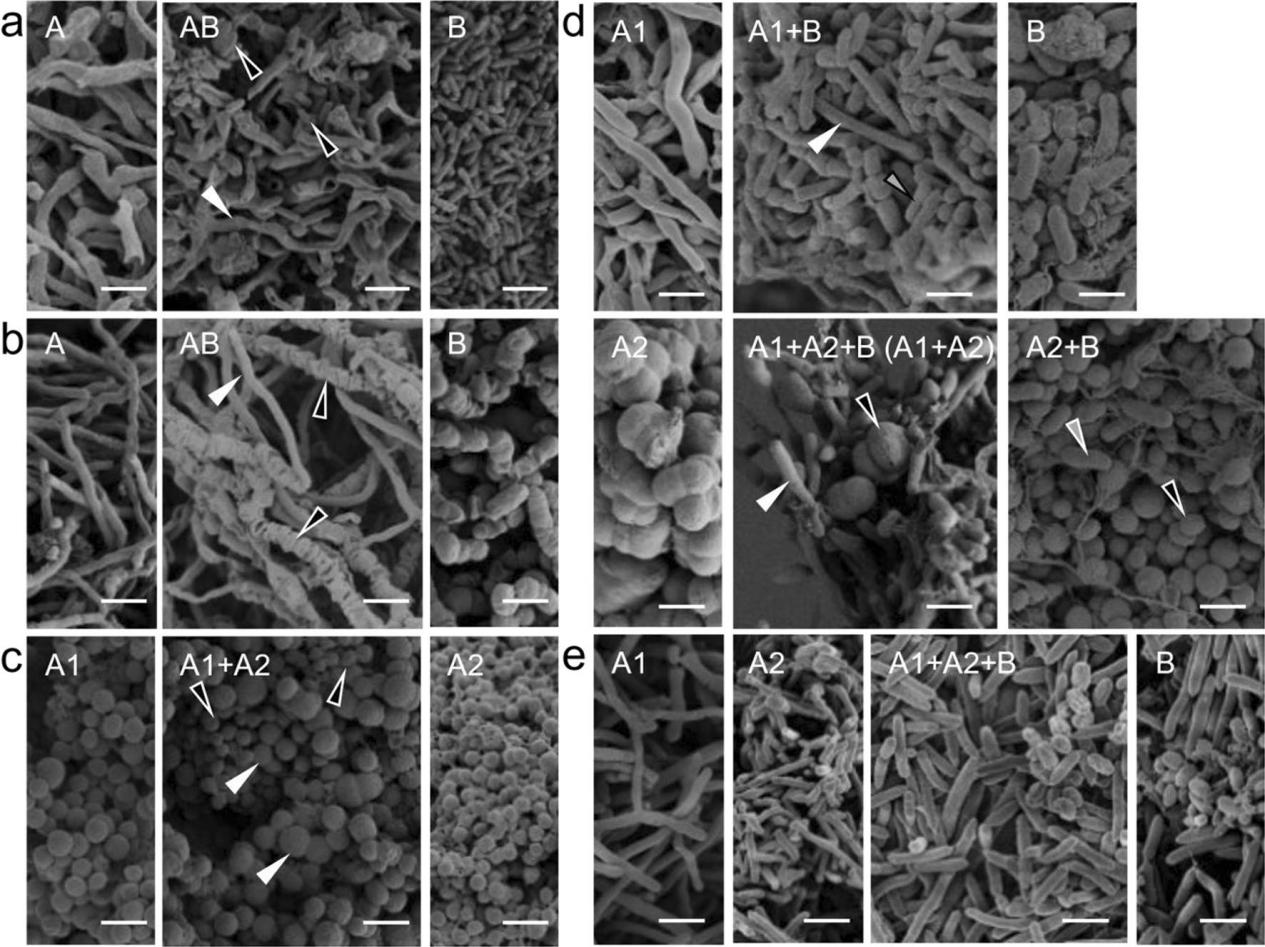
Scanning electron microscope image of co-isolated filamentous or cocci-shaped actinomycetes and other bacteria in co-culture. (**a**) Left image: monoculture of HEK314A. Middle image: co-culture of HEK314A/B. Right lower image: monoculture of HEK314B. White arrow indicates HEK314A filamentous cell and blank arrow indicates HEK314B bacilli-shaped cell. (**b**) Left image: monoculture of HEK830A. Middle image: co-culture of HEK830A/B. Right image: monoculture of HEK830B. White arrow indicates HEK830A filamentous cell and blank arrow indicates HEK830B bacilli-shaped cell. (**c**) Left image: monoculture of HEK654A1. Middle image: co-culture of HEK654A1/A2. Right image: monoculture of HEK654A2. White arrow indicates HEK654A1 large cocci-shaped cell and blank arrow indicates HEK654A2 small cocci-shaped cell. (**d**) Upper left image: monoculture of HEK652A1. Upper middle image: co-culture of HEK652A1/B. Upper right image: monoculture of HEK652B. Lower left image: monoculture of HEK652A2. Lower middle image: co-culture of HEK652A1/A2/B. Lower right image: co-culture of HEK652A2/B White arrow indicates HEK652A1 cell, blank arrow indicates HEK654A2 cell and gray arrow indicates HEK652B cell. (**e**) Left image: monoculture of HEK282A1. Left middle image: monoculture of HEK282A2. Middle image: co-culture of HEK282A1/A2/B. Right image: monoculture of HEK282B. (Scale bar = 2 µm).

HEK314 contained *Streptomyces drozdowiczii* HEK314A and Gram-negative *Sphingomonas oligoaromativorans* HEK314B. *Sph. oligoaromativorans* HEK314B had bacilli-shaped cells and the filamentous actinomycete *S. drozdowiczii* HEK314A had mycelial cells. Bacilli-shaped cells were found to attach on the mycelium of *S. drozdowiczii* HEK314A in co-culture, indicating that they formed co-aggregates (Fig. 3a).

HEK830 contained *Streptomyces tsukubensis* HEK830A and *Peribacillus simplex* HEK830B. *P. simplex* HEK830B had bacilli-shaped cells with a wrinkled cell surface, and it was found with filamentous actinomycete *S. tsukubensis* HEK830A mycelium in co-culture, indicating that they formed co-aggregates (Fig. 3b).

HEK654 contained the cocci-shaped actinomycetes *Kineococcus mangrovie* HEK654A1 and *Pseudokineococcus marinus* HEK654A2. Both cells were observed with cocci-shaped cells but the cell sizes were different in monocultures. *K. mangrovie* HEK654A1 cells were relatively large (1.0 µm) whereas *P. marinus* HEK654A2 cells were smaller (0.65 µm). Cells with both different sizes were observed in the cell pellet of the co-culture, indicating that they formed co-aggregates (Fig. 3c).

HEK652 contained *Streptomyces termitum* HEK652A1, actinomycetes *Quadrisphaera granulorum* HEK652A2, and Gram-negative *Methylobacterium phyllosphaerae* HEK652B. In monoculture, *S. termitum* HEK652A1 was observed in mycelium form, *Q. granulorum* HEK652A2 cells were cocci in tetrads, and *M. phyllosphaerae* HEK652B cells were bacilli (Fig. 3d). In co-culture of the three bacteria, cocci-shaped cells of *Q. granulorum* HEK652A2 were observed with mycelium of *S. termitum* HEK652A1, but bacilli-shaped cells were not observed. In co-culture of two bacteria, mycelium of *S. termitum* HEK652A1 was observed with bacilli-shaped cells of *M. phyllosphaerae* HEK652B, and *Q. granulorum* HEK652A2 was observed with bacilli-shaped cells of *M. phyllosphaerae* HEK652B. We observed that two of the three strains in this mixture could form coaggregates, but the three bacteria together did not form a single aggregate (Fig. 3d).

HEK282 contained *Streptomyces alboflavus* HEK282A1, actinomycetes *Cellulosimicrobium funkei* HEK282A2, and *Paenibacillus chitinolyticus* HEK282B. *S. alboflavus* HEK282A1 was observed in mycelium form. *C. funkei* HEK282A2 was observed as bacilli-shaped cells but also with significantly elongated cell shape. *P. chitinolyticus* HEK282B was also observed as bacilli-shaped cells with significantly elongated cell shape. Bacilli-shaped cells and filamentous cells were observed in co-culture but it was not possible to discriminate the origin from the SEM image (Fig. 3e). Therefore, we could not conclude whether those pairs formed mixed aggregates.

HEK332 and HEK423 each contained two *Streptomyces* species. Because they both had filamentous cell shape, it was not possible to discriminate between them by SEM (Supplemental Fig. 3). Therefore, we could not conclude whether those pairs formed mixed aggregates.

### Combined-culture of a co-isolated pair of actinomycete and MACB

**Figure 4:**
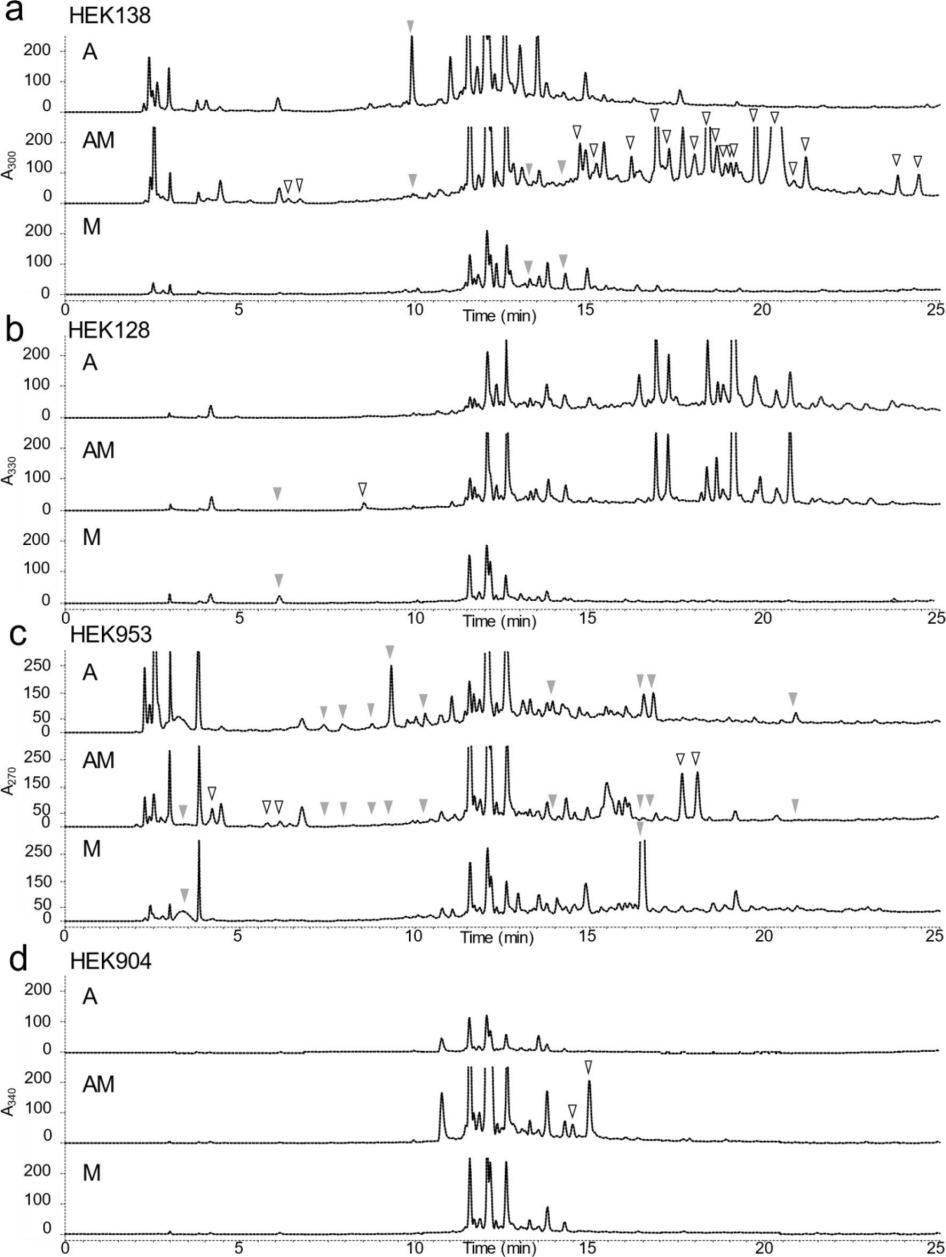
Metabolites analyses of naturally co-isolated bacterial pair in combined-culture using high performance liquid chromatography (HPLC). Blank arrows indicate the new peaks and gray arrows indicate the lost peaks that were observed consistently in all three replicates of combined-culture. (**a**) HPLC profiles for HEK128A and HEK128M monitored at 330 nm. (**b**) HPLC profiles for HEK138A and HEK138M monitored at 300 nm. (**c**) HPLC profiles for HEK904A and HEK904M monitored at 340 nm. (**d**) HPLC profiles for HEK953A and HEK953M monitored at 270 nm. The wavelengths monitored were selected to show the clear differences between combined-culture and monoculture.

**Figure 5:**
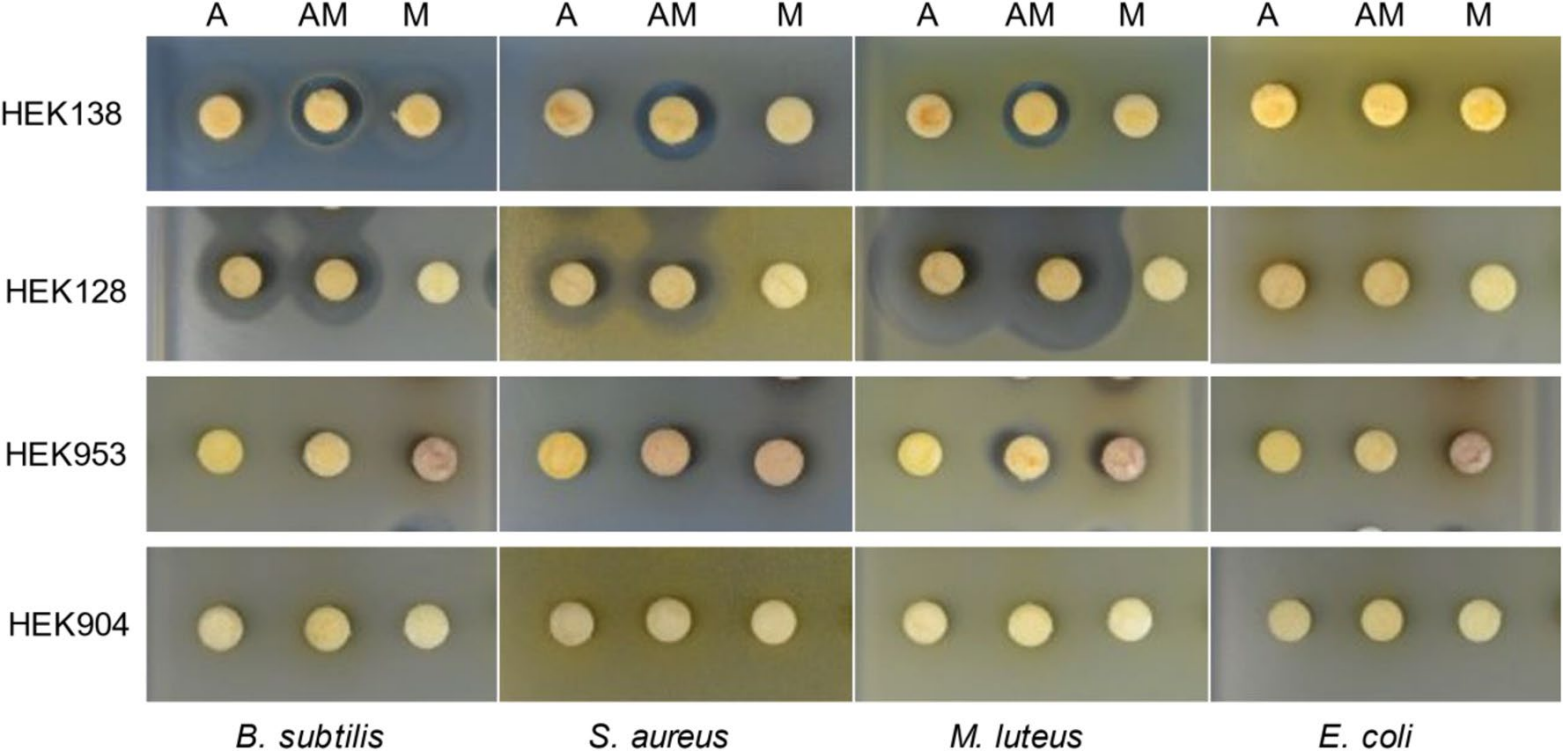
Paper disc assay. Clearance zones of Gram-positive *Bacillus subtilis* ATCC6633, *Staphylococcus aureus* 209PJC-1, *Micrococcus luteus* ATCC9341, and Gram-negative *Escherichia coli* NIHJ-2 were determined in response to extracts from culture of the natural pairs in combined-culture. (A: actinomycetes, M: MACB).

Because MACB can affect the secondary metabolites profile of actinomycetes, we compared the metabolites of monoculture and combined-culture using high performance liquid chromatography with diode array detector (PDA-HPLC). We found significant differences in metabolites extracted from the culture after combined-culture of the natural pair of actinomycetes and MACB when compared with monoculture (Figs. 4 and 5). Focusing on newly generated peaks, we found 19 new peaks with absorbance at 300 nm generated during combined-culture of HEK138A and HEK138M (Fig. 4a). Paper disk assays were performed^33^, and specific growth inhibition zones were observed for combined-culture extracts against tested Gram-positive bacteria *B. subtilis*, *Staphylococcus aureus*, and *Micrococcus luteus* (Fig. 5, Table 2) and translucent inhibition zones against Gram-negative *Escherichia coli*. The active compound against *E. coli* that gave a translucent inhibition zone which was still distinguishable but less transparent than the general inhibition zone was identified as desferrioxamine E by activity guided purification (Supplemental Fig. 9). For HEK128A and HEK128M, one new peak with absorbance at 330 nm was generated during combined-culture (Fig. 4b). Antibacterial activity was slightly increased by combined-culture (Fig. 5, Table 2). For HEK953A and HEK953M, five new peaks with absorbance at 270 nm were generated during combined-culture (Fig. 4c). Antibacterial activity was slightly decreased by combined-culture in this case (Fig. 5, Table 2). For HEK904A and HEK904M, two new peaks with absorbance at 340 nm were generated by combined-culture (Fig. 4d). Antibacterial activity was not observed in these strains (Fig. 5, Table 2).

**Table 2:** Paper disc assay.

	*B. subtilis*	*S. aureus*	*M. luteus*	*E. coli*
**HEK138**				
A	0	0	0	0
AM	◎15	◎13.8	◎12.3	◎14*
M	0	0	0	0
**HEK128**				
A	19	16.8	30	0
AM	◎19.5	◎17.2	30	0
M	0	0	0	0
**HEK953**				
A	0	0	0	0
AM	9.5	10.1	14	0
M	10.6	11.3	14.5	0
**HEK904**				
A	0	0	0	0
AM	0	0	0	0
M	0	0	0	0

Clearance zones of Gram-positive *Bacillus subtilis* ATCC6633, *Staphylococcus aureus* 209PJC-1, *Micrococcus luteus* ATCC9341, and Gram-negative *Escherichia coli* NIHJ-2 were determined in response to extracts from culture of the natural pairs in combined-culture. *Indicates the translucent inhibition zone. Double circles indicate the extracts with specific or improved activity by combined-culture. (number = growth inhibition zone, mm).

### Re-isolation of co-aggregated cells formed by actinomycetes and MACB

**Figure 6:**
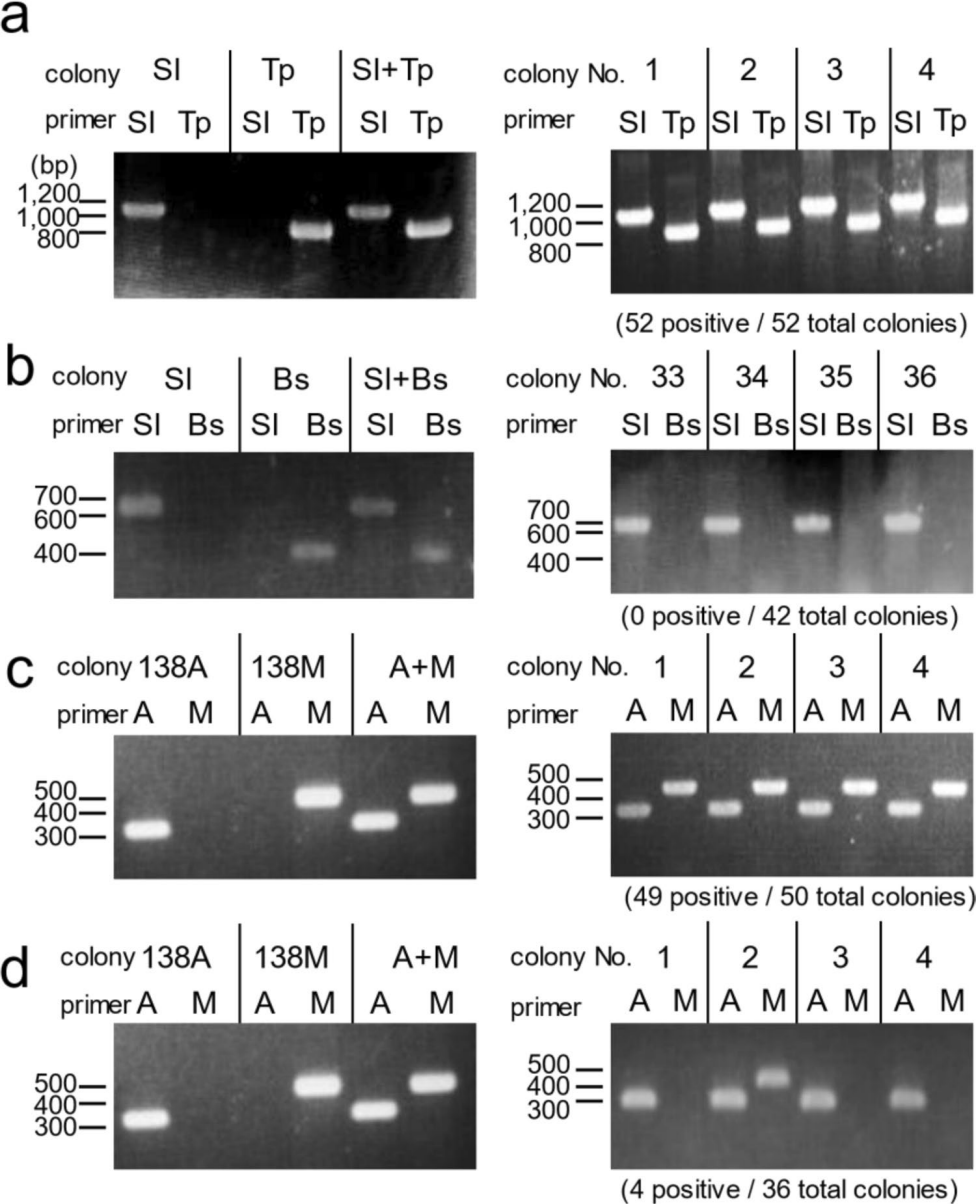
Detection of strains in co-isolated colony by selective PCR. Left side gel images are positive controls using a mixture of both cultures. Right side gel images are the results of colony PCR (all results are shown in Supplemental Figs. 1–4) (**a**) Selective colony PCR for detection of *Streptomyces lividans* (Sl) and *Tsukamurella pulmonis* (Tp) in isolated colonies. Colonies containing Tp/total colonies = 52/52 (100%). Original gel images are presented in Supplementary Fig. 4. (**b**) Selective colony PCR for detection of *S. lividans* (Sl) and *B. subtilis* (Bs) in isolated colonies. Colonies containing Tp/total colonies = 0/42 (0%). Original gel images are presented in Supplementary Fig. 5. (**c**) Selective colony PCR for detection of *Streptomyces* sp. HEK138A (138A) and *Mycolicibacterium* sp. HEK138M (138 M) in isolated colonies. Colonies containing 138 M/total colonies = 49/50 (98%). Original gel images are presented in Supplementary Fig. 6. (**d**) Selective colony PCR for detection of *Streptomyces* sp. HEK138A (138A) and *Mycolicibacterium* sp. HEK138M (138 M) in isolated colonies from soil. Colonies containing 138 M/total colonies = 4/36 (11%). Original gel images are presented in Supplementary Fig. 7.

To confirm the morphology of colonies formed by the mixed cultures of *Streptomyces* species and MACB, we performed a co-isolation experiment to test whether co-aggregated bacteria could be co-isolated in what looks like a single colony (see “Materials and methods” for details and Supplemental Fig. 2a). After liquid combined-culture, we isolated the pellets and incubated them after washing. *S. variegatus* HEK138A (Sv) and *M. septicum* HEK138M (Ms) combined-culture was compared with Sv and *Bacillus subtilis* (Bs) co-culture as negative control. The identity of isolates was confirmed by selective colony PCR. Sv + Ms showed significant co-isolation rate (49/50 colonies from isolated Sv pellets; Fig. 6c, Supplemental Fig. 6) comparable to the results of Sl + Tp (50/50 colonies from isolated Sl pellets; Fig. 6a, Supplemental Fig. 4). Because Bs was killed by co-culture with Sv (probably by the antibiotic produced by Sv; Table 2), we could not obtain the co-isolation rate for Sv + Bs (data not shown). Therefore, to confirm that non-associated pair cannot be co-isolated in this experiment, we used combination of Sl and Bs as negative control. In the negative control, pellets from co-culture of Sl + Bs did not show significant co-isolation rate (0/42 colonies from isolated Sv pellets; Fig. 6b, Supplemental Fig. 5). Taken together with the SEM images, these results confirmed that significant cell attachment occurred for *Streptomyces* species and MACB when compared with *B. subtilis*.

We further tested whether the co-aggregated pair could be co-isolated using the isolation protocol performed initially for the bacterial isolation from Hegura Island environmental samples (see “Materials and methods” for details). After the combined-culture, the culture was washed twice with phosphate buffered saline (PBS) and mixed with soil before isolation (Supplemental Fig. 2b). The results of Sv + Ms combined-culture, which was verified by selective colony PCR, showed several co-isolated colonies (4/36 colonies from isolated Sv pellets; Fig. 6d, Supplemental Fig. 7). These results suggested that a low proportion of co-aggregated cells could be stochastically co-isolated using the isolation protocol.

## Discussion

Mixing actinomycetes and MACB in the same laboratory culture resulted in a high chance of alterations in secondary metabolism. The actinomycetes and MACB are occasionally found to form co-aggregates when cultured together in the same laboratory culture. This study was initiated by an incidental finding of mixed culture stocks containing actinomycetes and MACB, which were isolated from colonies that appeared to be formed by single bacteria. Actinomycetes and MACB are ubiquitous soil bacteria, therefore it was speculated that this characteristic interaction takes place in the natural soil environment. Therefore, in this paper, we focused on the interaction between the naturally isolated pair of actinomycetes and MACB in terms of morphology and metabolic changes.

We collected 71 environmental samples from Hegura Island and obtained 1041 spore/cell bacterial stock cultures. Partial 16S rRNA gene sequencing of 182 strains identified 32 genera among these bacterial isolates, indicating that the environmental samples from Hegura Island contain a rich diversity of bacteria. Within the 1041 stocks, we found at least 11 stocks that contained more than two different strains. All co-isolated bacteria from those 11 stocks could grow in monoculture on general agar medium, indicating that there was no obligatory symbiotic relationship between the individual isolates. Taxonomic analysis based on 16S rRNA genes revealed that the co-isolated strains were not bacteria with characteristic properties but were ubiquitous soil bacteria. This suggested that soil bacteria associate with each other in the natural soil habitat and can thus be co-isolated. Interestingly, besides actinomycetes and MACB, other co-isolated bacterial pairs generated co-aggregates in liquid culture. This indicated that the physical association of bacteria does not require special properties but is facilitated by general interaction in the soil microbiome.

Actinomycetes are relatively dominant species in the rhizosphere^34^. Within the 11 stocks, seven mixed stocks were obtained from rhizosphere soil samples from different locations. This might suggest the relatively frequent association of bacteria with other bacteria in those habitats. Interspecies co-aggregation facilitates bacterial communication over physically short distances^35^. Several strategies are known for bacterial communication such as using small signaling molecules^36^, membrane vesicles^37,38^, and cell–cell physically-dependent communication over short distances^39,40^, including interspecies communication^41^. The interactions observed by SEM imaging did not provide evidential proof of known bacterial communication strategies. The ecological benefit of this association requires further study because there could be strong competition for nutrients and other impacts among cells within co-aggregates.

It was suggested that the co-aggregation between actinomycetes and MACB is a naturally occurring association with tight interaction and thus they were isolated together. Thus, we tested whether a co-aggregated bacterial pair could be isolated by forming a single colony. Cell pellets of actinomycetes grown in liquid culture were isolated and incubated on agar plates after washing. The growing colony appeared to be derived from a single strain of actinomycete. However, selective colony PCR indicated that the colony harbored both actinomycetes and MACB. When the cell pellets were isolated by a procedure similar to that of the original isolation from soil, 11.1% (4/36) of colonies were formed by two strains. This demonstrated that co-aggregating strains could be co-isolated under these conditions.

We tested the effect of combined-culture on metabolic changes in the co-isolated pairs. In our previous work using the culture collection obtained from Hegura Island, we isolated natural products from combined-culture with *T. pulmonis* such as: streptoaminals and 5-alkyl-1,2,3,4-tetrahydroquinolines from *Streptomyces nigrescens* HEK616^13,14^, dracolactams from *Micromonospora wenchangensis* HEK797^12^, and streptogramins from *Streptomyces albogriseolus* HEK740^42^. Therefore we asked whether the original combination of actinomycetes and MACB affects their metabolism. As expected, we observed significant differences in the HPLC chromatograms of extracts from combined-culture when compared with monoculture of the naturally isolated pair. The extracted metabolites of HEK138A and HEK138M showed specific growth inhibition activity indicating potential antibacterial activity. Antibiotic production by the actinomycete (HEK138A) would be beneficial for the MACB (HEK138M), providing protection (by antibiotic) to secure the natural habitat without killing each other. MACB (e.g., *Rhodococcus* sp.) have a degradative role in the environment, which might provide easily available nutrient sources for the actinomycete (HEK138A). There are no reports to date of the isolation of natural products from *Isoptericola* species (HEK904A), therefore identification of induced metabolites in combined-culture of HEK904A and M would be interesting for future analysis. Taken together, the approach of co-culturing naturally isolated bacterial co-aggregates could be an option for screening new bioactive secondary metabolites.

## Materials and methods

### Isolation of actinomycetes

We collected 71 environmental samples from Hegura Island in June 2008 (Supplemental Table 5 and Fig. 10). Soils from 3 to 5 cm depth were collected into sterilized 50 ml conical tubes using autoclaved stainless spoons (spatulas). Briefly, samples were suspended in sterilized ddH_2_O and divided in half for phenol or water treatments. Phenol solution (150 mg/l) was added to the one half of the suspension to kill phenol-sensitive bacteria. Subsequently the phenol-treated suspension was spread with appropriate dilution on ISP4 agar or humic acid agar with antifungal antibiotics. Ten milliliters of sterilized ddH_2_O was added to 1 g of environmental sample and suspended with agitation (on a shaker at 300 rpm) for 10 min. The suspended solution was further diluted by addition of 9 ml of sterilized ddH_2_O. In the next step, 9 ml of sterilized ddH_2_O or phenol suspension (1 g/140 ml) was added to the 1 ml of diluted suspension and mixed for 10 min (on a shaker at 300 rpm). The bacteria were grown on ISP4 medium or humic acid medium at 30 °C for 7–10 days. ISP4 agar medium contained soluble starch (1.0%), (NH_4_)_2_SO_4_ (0.2%), CaCO_3_ (0.2%), K_2_HPO_4_ (0.1%), MgSO_4_⋅7H_2_O (0.1%), NaCl (0.1%), and trace salt solution (0.1% v/v) containing FeSO_4_⋅7H_2_O (0.1%), MnCl_2_⋅4H_2_O (0.1%), ZnSO_4_⋅7H_2_O (0.1%), and agar (2.0%) in distilled water (pH 7.2). Humic acid agar medium contained humic acid (0.1%), Na_2_HPO_4_ (0.05%), KCl (0.171%), MgSO_4_⋅7H_2_O (0.005%), FeSO_4_⋅7H_2_O (0.001%), CaCO_3_ (0.001%), B-vitamins (0.0005% of each except for 0.0025% biotin), in distilled water (pH 7.2). To prevent fungal growth, amphotericin B (final conc. 20 µg/ml) and cycloheximide (final conc. 20 µg/ml) were dissolved in dimethyl sulfoxide (DMSO) and added to the agar medium after autoclaving.

Under the stereomicroscope, the apparent single colonies forming on ISP4 or humic acid agar plates were picked by toothpick and sub-cultured on new ISP4 or humic acid agar plates. After subculture, we checked for contamination by other bacteria in this process. The subcultured actinomycetes that appeared as single bacteria were further subcultured on new ISP4 or humic acid agar plates and incubated for 7–10 days. Grown cells or matured spores were recovered from the agar plates to make 20% glycerol spore/cell stocks and stored at − 80 °C. A total of 1041 spore/cell stocks were obtained by these isolation processes.

### 16S rRNA gene sequencing

Genomic DNA of the isolated bacteria was extracted and purified using the cetyltrimethylammonium bromide method^43^. 16S rRNA genes of the isolated bacteria were amplified by PCR using a thermal cycler (ProFlexTM PCR system, Applied Biosystems). Universal primers (16S-F:5ʹ-GAGAAGCTTAGAGTTTGATCCTGGCTCAG-3ʹ and 16S-R5ʹ-GAGAAGCTTAC-GGCTACCTTGTTACGACT-3ʹ) and GoTaq Green Master Mix (Promega Co.) were used for PCR by following the manufacturers protocol^44^. Sanger sequencing was performed at Eurofins Genomics Co. using the same primer sets. The partial 16S rRNA gene sequence data obtained was compared by nblast to identify the most similar strains in the National Center for Biotechnology Information (NCBI) database. We named the bacterial species in this study based on the closest identified relatives of the 16S rRNA gene sequences (if above 98%). Sequences of the 16S rRNA genes are registered in accession number of LC656369, LC656370, LC656371, LC656372, LC656373, LC656374, LC656375, LC656376, LC656377, LC656378, LC656379, LC656380, LC656381, LC656382, LC656383, LC656384, LC656385, LC656386, LC656387, LC656388, LC656389, LC656390, LC656391, LC656392.

### Construction of 16S rRNA gene phylogenetic tree

Partial 16S rRNA gene sequences were obtained from 182 culture stocks and used to construct a phylogenetic tree. 182 culture stocks were selected at random. ClustalW was used for the sequence alignment and the maximum likelihood method was used to generate the phylogenetic tree using MEGA X software. The phylogenetic analysis was tested using bootstrap values based on 1000 replications, and recommended parameters: the General Time Reversible model and Gamma Distributed with Invariant Site (discrete Gamma categories = 5). The 16S rRNA gene sequence from *Escherichia coli* K12 was used as outgroup, and manually deleted from the figure.

### Bacterial co-culture conditions

Bacteria were grown on ISP2 agar plates for 3–5 days. Each isolated actinomycete and MACB were cultivated separately in test tubes containing 10 ml of ISP2 medium^43^ (for morphological observation) or V-22 medium (for metabolite analysis) as seed culture. ISP2 medium contained glucose (0.4%), malt extract (1.0%, BD Biosciences), and yeast extract (0.4%, BD Biosciences) in distilled water (pH 7.2) and V-22 medium contained soluble starch (1.0%), glucose (0.5%), NZ-case (0.3%), yeast extract (0.2%), Bacto tryptone (0.5%), K_2_HPO_4_ (0.1%), Mg⋅7H_2_O (0.05%), and CaCO_3_ (0.3%) in distilled water (pH 7.0). The test tubes were shaken on a rotary shaker (180 rpm) at 30 °C for 2 days. Broth containing the actinomycete (1 ml) and the MACB (1 ml) were transferred simultaneously into 100 ml flasks containing 20 ml of YGGS medium (described below) for morphological observation and cultured on a rotary shaker (180 rpm) at 30 °C for 5 days. For the metabolite analysis, 1 ml of actinomycetes broth and 1 ml of MACB broth were transferred simultaneously into 500 ml K^−1^ flasks containing 100 ml of A-3 M production medium and cultured on a rotary shaker (200 rpm) at 30 °C for 7 days. A-3 M medium contained glucose (0.5%), soluble starch (2.0%), glycerol (2.0% w/v), pharmamedia (cottonseed meal, 1.5%), and yeast extract (0.3%, BD Biosciences) in distilled water (pH 7.2). YGGS was A-3 M medium without pharmamedia.

### Microscopy

Samples for SEM were prepared as follows. One milliliter of liquid culture was collected into a 1.5 ml tube. After centrifugation (3000 g, 1 min) and removal of the supernatant, 1 ml of 2.5% glutaraldehyde in 0.1 M sodium phosphate buffer (pH 7.4) was added to the cell pellets and incubated for 1 h at 4 °C. After discarding the solution, 1 ml of 0.1 M sodium phosphate buffer (pH 7.4) was added to rinse the sample. Prior to establishing the current method using a centrifuge, we tested isolates aggregation by using combination of *B. subtilis* and *S. lividans* TK23 as control. We did not observe the aggregated pellet of *S. lividans* TK23 with *B. subtilis* by method using centrifugation. (data not shown) Then, 1% OsO4 in 100 mM sodium phosphate buffer (pH 7.4) was added and incubated for 1 h on ice. After discarding the solution, 1 ml of 0.1 M sodium phosphate buffer (pH 7.4) was added to rinse the sample. Subsequently, the sample was dehydrated by stepwise addition of 50%, 70%, 90%, and 100% ethanol (1 ml each), and finally substituted by t-butyl alcohol, and stored at 4 °C until use. The sample was dried under vacuum before Pt/Pd sputter coating (E-1030, Hitachi, Japan) and observation by SEM (S-4800, Hitachi, Japan). Stereomicroscope images of bacterial colonies formed on agar plates were obtained by Nikon SMZ25 equipped with a SHR Plan Apo 1.6 × lens (Nikon) and DS-Ri2 digital camera (Nikon).

### Bacterial co-isolation assay

*S. lividans* (Sl), *T. pulmonis* (Tp), HEK138A, HEK138M, and *B. subtilis* (Bs) were inoculated into 10 ml of ISP2 medium and precultured for 2 days at 180 rpm and 30 °C. The following ratios of aliquot from preculture were used to inoculate 20 ml of YGGS medium in a 100 ml Erlenmeyer flask for co-culture (Sl : Tp = 1 ml : 300 µl, Sl : Bs = 1 ml : 300 µl, 138A : 138 M = 1 ml : 300 µl, 138A : Bs = 1 ml : 300 µl). After culture for 5–6 days (180 rpm, 30 °C), 1 ml of culture was used for further analysis. First, the whole culture broth was passed through a stainless-steel sieve (φ125 µm, 7.5 × 2.0 cm, SANPO Co.) to trap the cell pellets. Then 500 ml of PBS buffer was used to thoroughly wash the bacterial pellets that were trapped in the sieve. Subsequently, the bacterial pellets were recovered from the sieve and the washing step was repeated. After the washing step, the bacterial pellets were transferred to the center of an ISP4 agar plate and further incubated for 7 days. The growing colony images were obtained by stereomicroscope and the primers (see Supplemental data) were used to amplify specific regions of the 16S rRNA gene by PCR using GoTaq Green master mix (Promega). Agarose gel (1.5% w/v) was used for DNA electrophoresis.

### Re-isolation of co-aggregated cells from soil

HEK138A or HEK138M were inoculated in 10 ml ISP2 medium and precultured for 2 days (180 rpm, 30 °C). The following ratio of aliquot from preculture was used to inoculate 20 ml of YGGS medium in a 100 ml Erlenmeyer flask for combined-culture (HEK138A : HEK138M = 1 ml : 300 µl). After culture for 5 days (180 rpm, 30 °C), the whole culture broth was transferred to a conical tube (50 ml) and left to stand for 2 min to allow pellet sedimentation. The upper layer was discarded and 20 ml of ddH_2_O was added to wash the cell pellets. This washing step was repeated twice. Then 1 g of autoclaved soil (obtained from Yayoi campus) was added to the collected cell pellets. After addition of soil, the process for bacterial isolation was repeated as described above. (see Supplemental data).

### Primer sequences used for specific detection in the bacterial co-isolation assay

For Sl and Tp combined-culture, the following primers were used to specifically amplify the partial 16S rRNA gene regions. (Sl: *S. lividans*, Tp: *T. pulmonis*) Sl-Fw: 5ʹ-GAACCACTTCGGTGGGGA-3ʹ, Sl-Rv: 5ʹ-CCAACACCCCGAAGGGCT-3ʹ, Tp-Fw: 5ʹ-TTCCCCTGCATGGGGGTT-3ʹ, Tp-Rv 5ʹ-AGAAGGCACAAGACAAACCGA-3ʹ. For Sl and Bs co-culture, the following primers were used to specifically amplify the partial 16S rRNA gene region. (Bs: *B. subtilis*) Sl-Fw: 5ʹ-CAGGCATCTGCGAGGTTC-3ʹ, Sl-Rv: 5ʹ-CACGGACAACGTGGAATGTTG-3ʹ, BsFw: 5ʹ-CAAGTGCCGTTCAAATAGG-3ʹ, Bs-Rv: 5ʹ-AGGGGCGGAAACCCCCTA-3ʹ. For Sv and Ms combined-culture, the following primers were used to specifically amplify the partial 16S rRNA gene region. (Sv: HEK138A, Ms: HEK138M) Sv-Fw: 5ʹ-GGGCAACATTCCACGTTG-3ʹ, Sv-Rv: 5ʹ-CATCACCCCGAAGGGCAT-3ʹ, Ms-Fw: 5ʹ-CGTTGTTCGTGAAAACTCACA-3ʹ, Ms-Rv: 5ʹ-GAACCAATATCTCTACTGGCG-3ʹ.

### Metabolite analysis by HPLC

Fifteen milliliters of culture broth was collected and an equal volume of butanol was added to extract the metabolites. After centrifugation, 12 ml of the butanol layer was collected and dried *in vacuo*. Dried samples were dissolved in 1.2 ml of DMSO (to concentrate the solution tenfold), placed on a 96 well plate (Agilent Technologies), and then covered with a silicone closing mat (Agilent Tech.). Metabolites were analyzed by PDA-HPLC (Agilent 1260 Infinity LC system, Agilent Tech.). Four microliters of sample was applied for each data acquisition. The HPLC system was equipped with a Cosmosil 5C18-AR-II column (4.6 i.d. × 250 mm, Nacalai Tesque). Acetonitrile and H_2_O (MilliQ) containing 0.1% formic acid was used as mobile phase. Acetonitrile at 5% was the mobile phase for the first 5 min, and then increased to 95% by linear gradient from 5 to 25 min, and then maintained at 95% until 30 min. The column temperature were kept at 40 °C and the flow was 1.0 ml/min.

### Paper-disc growth inhibition assay

*Bacillus subtilis* ATCC6633, *Staphylococcus aureus* 209PJC-1, *Micrococcus luteus* ATCC9341, and *Escherichia coli* NIHJ-2 were inoculated in 10 ml Mueller Hinton (MH) medium (BD Biosciences) and precultured for 1 day (180 rpm, 30 °C). Preculture (0.6 ml) was added to 6 ml of soft agar (1.6% nutrient broth (BD Biosciences), 0.5% agar). The solution was applied as an overlay to an agar plate (140 mm × 100 mm. Eiken chemical Co., LTD) containing 15 ml of MH agar. Twenty microliters of samples dissolved in DMSO (extracts from 0.2 ml culture) were transferred to paper discs (8 mm, ADVANTEC) and placed on an agar plate. After incubation at 30 °C for 1 day, the zones of growth inhibition were measured. The experiment was performed twice for the biological replicate. Gentamicin (5 µg) was used as positive control and DMSO (20 µL) was used as negative control.

## Supplementary Information

Supplementary Information.
