# Supplementary material for: Intimate relationships among actinomycetes and mycolic acid-containing bacteria
Source: Sci Rep. 2022 May 4;12:7222. doi: 10.1038/s41598-022-11406-2 (PMC9068768; doi:10.1038/s41598-022-11406-2)
Supplement: Supplementary file 1 — Supplementary Information. [file 41598_2022_11406_MOESM1_ESM.docx]

**Supplemental data**

**Title:**

Intimate relationships among soil bacteria: actinomycetes and mycolic acid-containing bacteria

**Authors:**

Manami Kato†,[a] Shumpei Asamizu†*,[a,b] and Hiroyasu Onaka* [a,b]

**Affiliation:**

[a] Graduate School of Agricultural and Life Sciences, The University of Tokyo, 1-1-1 Yayoi, Bunkyo, Tokyo 113-8657, Japan

[b] Collaborative Research Institute for Innovative Microbiology, The University of Tokyo 1-1-1 Yayoi, Bunkyo, Tokyo 113-8657, Japan

†These authors contributed equally to this work.

***Corresponding authors:**

Shumpei Asamizu, Email: asamizu@mail.ecc.u-tokyo.ac.jp

Hiroyasu Onaka, Email: aonaka@mail.ecc.u-tokyo.ac.jp

**Contents of supplemental data**

**Supplemental table 1**

Natural product discovery from combined culture.

**Supplemental figure 1**

Methods for bacterial isolation from Hegura Island environmental samples used in this study.

**Supplemental figure 2**

Methods to assay the bacterial coaggregation used in this study.

**Supplemental table 2**

Taxonomy of the 166 strains and definitions used in this study.

**Supplemental table 3**

List of strains in the combined clade shown in Figure 1.

**Supplemental figure 3**

Scanning electron microscope image of co-isolated filamentous or cocci-shaped actinomycetes and other bacteria in co-culture.

**Supplemental figure 4**

Analysis of the colonies obtained by co-isolation experiments from combined-culture of *Streptomyces lividans* TK23 and *Tsukamurella pulmonis* TP-B0596.

**Supplemental figure 5**

Analysis of the colonies obtained by co-isolation experiments from co-culture of *Streptomyces lividans* TK23 and *Bacillus subtilis* 168.

**Supplemental figure 6**

Analysis of the colonies obtained by co-isolation experiments from combined culture of *Streptomyces* sp. HEK138A and *Mycobacterium* sp. HEK138M.

**Supplemental figure 7**

Analysis of the colonies obtained by co-isolation experiments from combined culture of *Streptomyces* sp. HEK138A and *Mycobacterium* sp. HEK138M from soil.

**Supplemental figure 8**

HPLC profile of the metabolites from combined culture between the natural co-isolated pair.

**Supplemental figure 9**

Identification of active compound from HEK138A and M.

**Supplemental table 4**

Sources and methods for the initial isolation from the Hegura Island environmental samples of tested bacteria used in this study.

**Supplemental table 5**

Feature of collected environmental samples from the Hegura Island.

**Supplemental figure 10**

Sampling location and sampling points.

**References**

**Supplemental table 1.** Natural product discovery from combined culture. Natural products discovered from combined culture of actinomycetes with *Tsukamurella pulmonis* TP-B0596 (MACB) are listed in this table.

| strain | induced natural products | original producing strain | references |
| --- | --- | --- | --- |
| *Actinosynnema mirum* NBRC 14064 | mirilactams C-E |  | ^1^ |
| *Amycolatopsis* sp. 26-4 | amycolapeptins A and B |  | ^2^ |
| *Catenuloplanes* sp. RD067331 | catenulobactins A and B |  | ^3^ |
| *Micromonospora wenchangensis* HEK797 | dracolactams A and B |  | ^4^ |
| *Saccharothrix* sp. A1506 | saccharothriolide C-2 |  | ^5^ |
| *Streptomyces albogriseolus* HEK740 | streptogramin B and L-156,587 |  | ^6^ |
| *Streptomyces cinnamoneus* NBRC 13823 | arcyriaflavin E |  | ^7^ |
| *Streptomyces davawensis* JCM 4913 | desferrioxamine derivatives |  | ^8^ |
| *Streptomyces endus* S-522 | alchivemycin A and B |  | ^9^ |
| *Streptomyces nigrescens* HEK616 | streptoaminals |  | ^10, 11^ |
|  | 5-alkyl-1,2,3,4-tetrahydroquinolines |  | ^10, 12, 13^ |
| *Streptomyces* sp. CJ-5 | chojalactones A-C |  | ^14^ |
| *Streptomyces* sp. KUSC_F05 | longicatenamides A-D |  | ^15^ |
| *Streptomyces* sp. NZ-6 | niizalactams A-C |  | ^16^ |
| *Umezawaea* sp. RD066910 | umezawamides |  | ^17^ |
| heterologous expression in *S. lividans* TK23 | goadsporin A-C | from *Streptomyces* sp. TP-A0584 | ^18, 19^ |
| heterologous expression in *S. lividans* TK23 | staurosporin | from *Streptomyces* sp. TP-A0274 | ^19^ |
| heterologous expression in *S. lividans* TK23 | rebeccamycin | from *Lechevalieria aerocolonigenes* ATCC 39243 | ^19^ |
| heterologous expression in *S. lividans* TK23 | streptoaminals | from *Streptomyces nigrescens* HEK616 | ^10^ |
| heterologous expression in *S. lividans* TK23 | 5-alkyl-1,2,3,4-tetrahydroquinolines | from *Streptomyces nigrescens* HEK616 | ^10^ |


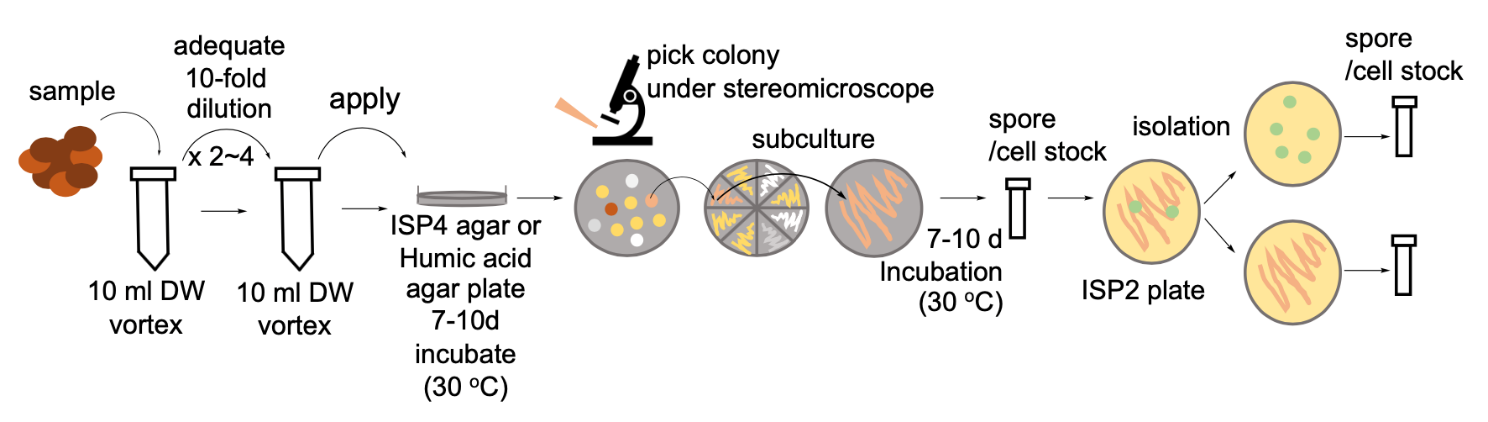
**Supplemental figure 1**

**Methods for bacterial isolation from Hegura Island environmental samples used in this study.** General dilution methods were used in this study.


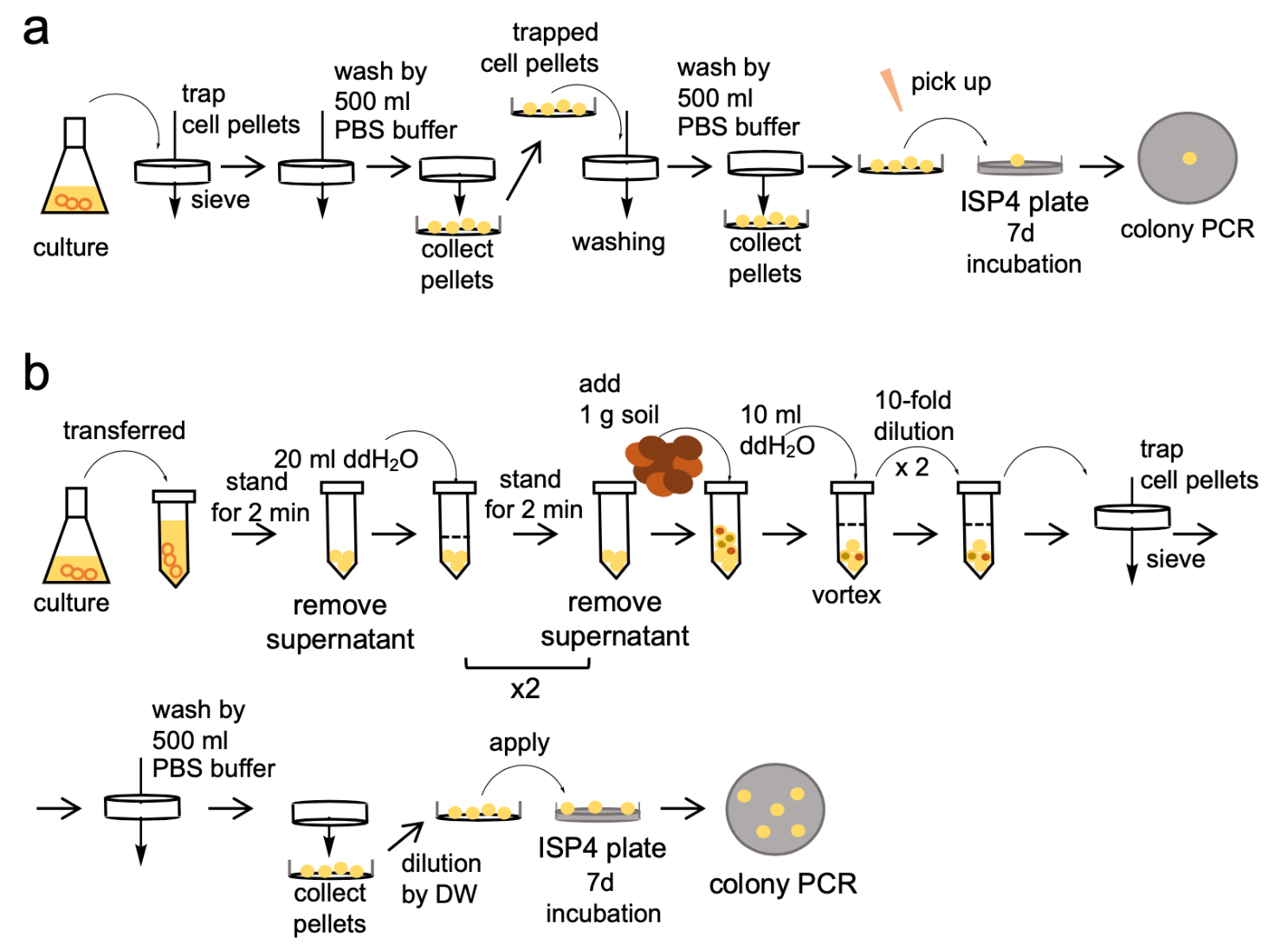
**Supplemental figure 2**

**Methods to assay the bacterial coaggregation used in this study.** (a) Cell pellets from liquid co-culture were captured by stainless-steel sieve and washed with phosphate-buffered saline to remove planktonic cells. Then the captured cell pellets were picked up by tweezers and inoculated on the agar medium to confirm that the cell pellet contained both filamentous actinomycetes and mycolic acid-containing bacteria. (b) Cell pellets form liquid co-culture were washed with ddH_2_O twice to remove excess planktonic cells and collected sedimented cells were suspended with sterilized soil. Cells suspended in soil were diluted in ddH_2_O and pellets were captured by stainless-steel sieve. Then the captured cell pellets were diluted in ddH_2_O and inoculated on the agar medium.

Ten milliliters of sterilized ddH_2_O was added to the 1 g of sample and suspended with agitation (on a shaker at 300 rpm) for 10 min. The suspended solution (1 ml) was further diluted by addition of 9 ml of sterilized ddH_2_O and suspended with agitation for 10 min (on a shaker at 300 rpm). Then, the solution was passed through a stainless-steel sieve to trap the pellet, and 500 ml of PBS buffer was additionally passed to thoroughly wash the bacterial pellets that were trapped in the sieve. This step was necessary to remove excess cells of HEK138M prior to inoculation on the agar plate. Subsequently, the bacterial pellets were recovered from the sieve and suspended in ddH_2_O. Cell suspension (100 µL) was inoculated onto an ISP4 agar plate and further incubated at 30°C for 7 days. The images of growing colonies were obtained by stereomicroscope, and selective PCR was performed to determine the strain content of the growing colonies.

**Supplemental table 2.** Taxonomy of the 182 isolated strains from Hegura Island based on partial 16S rRNA gene sequences and definitions used in this study.

| phylum | class | order | family | genus | number of strains | definition used in this study |
| --- | --- | --- | --- | --- | --- | --- |
| Actinobacteria | Actinomycetia | Corynebacteriales | Nocardiaceae | Rhodococcus | 4 | MACB |
|  |  |  |  | Nocardia | 3 |  |
|  |  |  | Gordoniaceae | Gordonia | 2 |  |
|  |  |  | Tsukamurellaceae | Tsukamurella | 1 |  |
|  |  |  | Mycobacteriaceae | Mycolicibacterium | 1 |  |
|  |  | Glycomycetales | Glycomycetaceae | Glycomyces | 1 | filamentous/coccal actinomycetes |
|  |  | Kineosporiales | Kineosporiaceae | Kineococcus | 2 |  |
|  |  |  |  | Quadrisphaera | 1 |  |
|  |  |  |  | Pseudokineococcus | 1 |  |
|  |  | Micrococcales | Microbacteriaceae | Microbacterium | 4 |  |
|  |  |  |  | Agromyces | 3 |  |
|  |  |  |  | Leifsonia | 1 |  |
|  |  |  | Promicromonosporaceae | Isoptericola | 3 |  |
|  |  |  |  | Cellulosimicrobium | 3 |  |
|  |  |  |  | Krasilnikoviella | 2 |  |
|  |  |  |  | Promicromonospora | 1 |  |
|  |  |  |  | Luteimicrobium | 1 |  |
|  |  |  | Cellulomonadaceae | Sediminihabitans | 1 |  |
|  |  | Micromonosporales | Micromonosporaceae | Micromonospora | 9 |  |
|  |  |  |  | Actinoplanes | 1 |  |
|  |  | Pseudonocardiales | Pseudonocardiaceae | Amycolatopsis | 3 |  |
|  |  |  |  | Saccharopolyspora | 1 |  |
|  |  |  |  | Saccharothrix | 1 |  |
|  |  | Streptomycetales | Streptomycetaceae | Streptomyces | 116 |  |
|  |  |  |  | Kitasatospora | 4 |  |
|  |  | Streptosporangiales | Thermomonosporaceae | Actinomadura | 2 |  |
|  |  |  | Streptosporangiaceae | Microbispora | 1 |  |
|  |  |  | Nocardiopsaceae | Nocardiopsis | 1 |  |
|  |  |  | Streptosporangiaceae | Nonomuraea | 1 |  |
| Proteobacteria | Alphaproteobacteria | Hyphomicrobiales | Devosiaceae | Devosia | 1 | other bacteria |
|  |  |  | Methylobacteriaceae | Methylobacterium | 4 |  |
|  |  |  | Rhizobiaceae | Rhizobium | 2 |  |

**Supplemental table 3.** List of strains in the combined clade shown in Figure 1.

| *Strepotmyces* spp. Group 1 | *Streptomyces* spp. Group 4 |
| --- | --- |
| *Streptomyces gilvifuscus* HEK217 | *Streptomyces lydicus* HEK497 |
| *Streptomyces rameus* HEK174 | *Streptomyces monticola* HEK574 |
| *Streptomyces fradiae* HEK219 | *Streptomyces sioyaensis* HEK934 |
| *Streptomyces neyagawaensis* HEK299 | *Streptomyces yogyakartensis* HEK417 |
| *Streptomyces bottropensis* HEK56 | *Streptomyces amphotericinicus* HEK747 |
| *Streptomyces scabies* HEK53 | *Streptomyces qinglanensis* HEK208 |
| *Streptomyces* spp. Group 2 | *Streptomyces ramulosis* HEK967 |
| *Streptomyces malachitospinus* HEK348 | *Streptomyces angustmyceticus* HEK277 |
| *Streptomyces violaceorubidus* HEK325 | *Streptomyces platensis* HEK633 |
| *Streptomyces tendae* HEK14 | *Microbacterium* spp. Group |
| *Streptomyces coelescens* HEK126 | *Microbacterium maritypicum* HEK898 |
| *Streptomyces tuirus* HEK58 | *Microbacterium paraoxydans* HEK943 |
| *Streptomyces olivaceus* HEK143 | *Microbacterium yannicii* HEK924 |
| *Streptomyces albogriseolus* HEK182 | *Microbacterium* testaceus HEK540 |
| *Streptomyces griseoflavus* HEK263 | *Agromyces* spp. Group |
| *Streptomyces iakyrus* HEK15 | *Agromyces allii* HEK874 |
| *Streptomyces ambofaciens* HEK180 | *Agromyces aureus* HEK999 |
| *Streptomyces nigra* HEK46 | *Agromyces hippuratus* HEK834 |
| *Streptomyces* spp. Group 3 | *Micromonospora* spp. Group |
| *Streptomyces badius* HEK513 | *Micromonospora harpali* HEK31 |
| *Streptomyces gelaticus* HEK253 | *Micromonospora spongicola* HEK82 |
| *Streptomyces atratus* HEK825 | *Micromonospora saelicesensis* HEK663-1 |
| *Streptomyces parvus* HEK402 | *Micromonospora humi* HEK441 |
| *Streptomyces sundarbansensis* HEK231 | *Micromonospora aurantiaca* HEK539 |
| *Streptomyces bacillaris* HEK211 | *Micromonospora coxensis* HEK204 |
| *Streptomyces californicus* HEK341 | *Micromonospora marina* HEK794 |
| *Streptomyces fulvissimus* HEK886 | *Micromonospora siamensis* HEK981 |
| *Streptomyces griseus* HEK950 |  |
| *Streptomyces flavogriseus* HEK308 |  |
| *Streptomyces baarnensis* HEK597 |  |
| *Streptomyces panaciradicis* HEK666 |  |
| *Streptomyces pratensis* HEK782 |  |

a


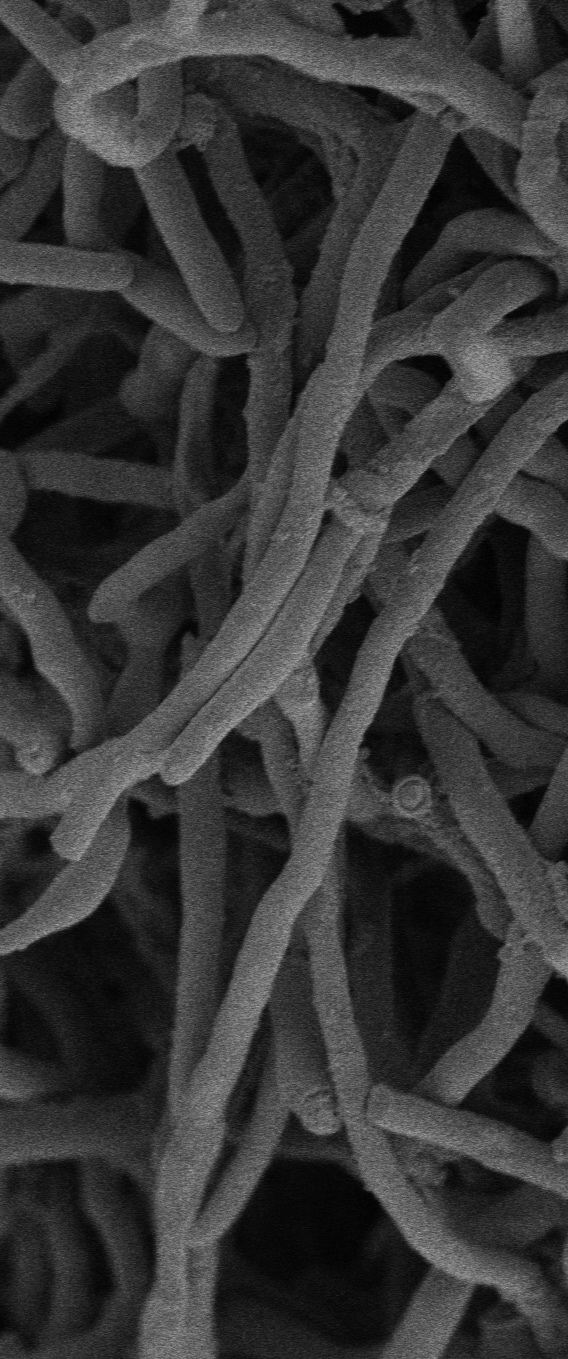


A1


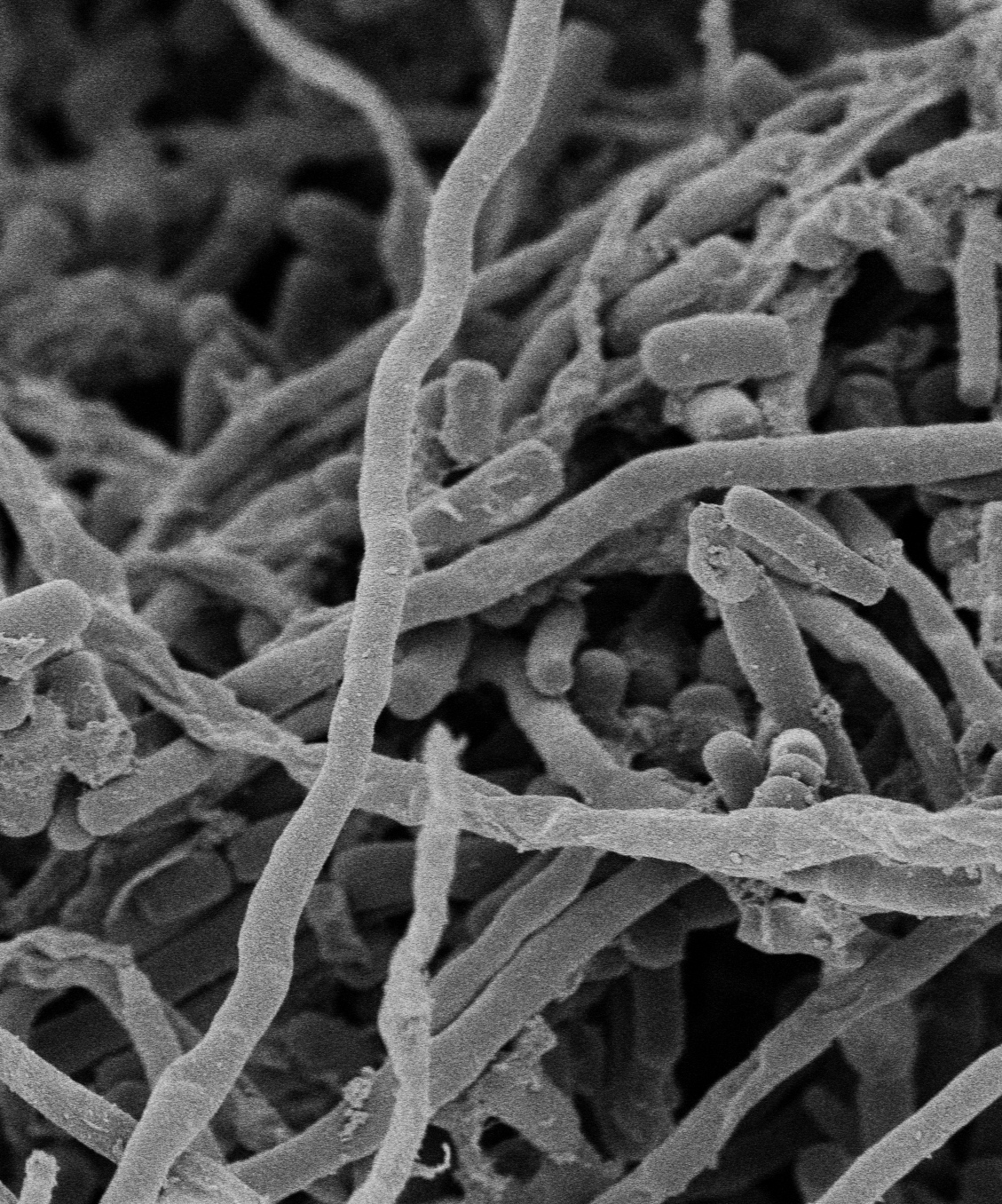


A1+A2


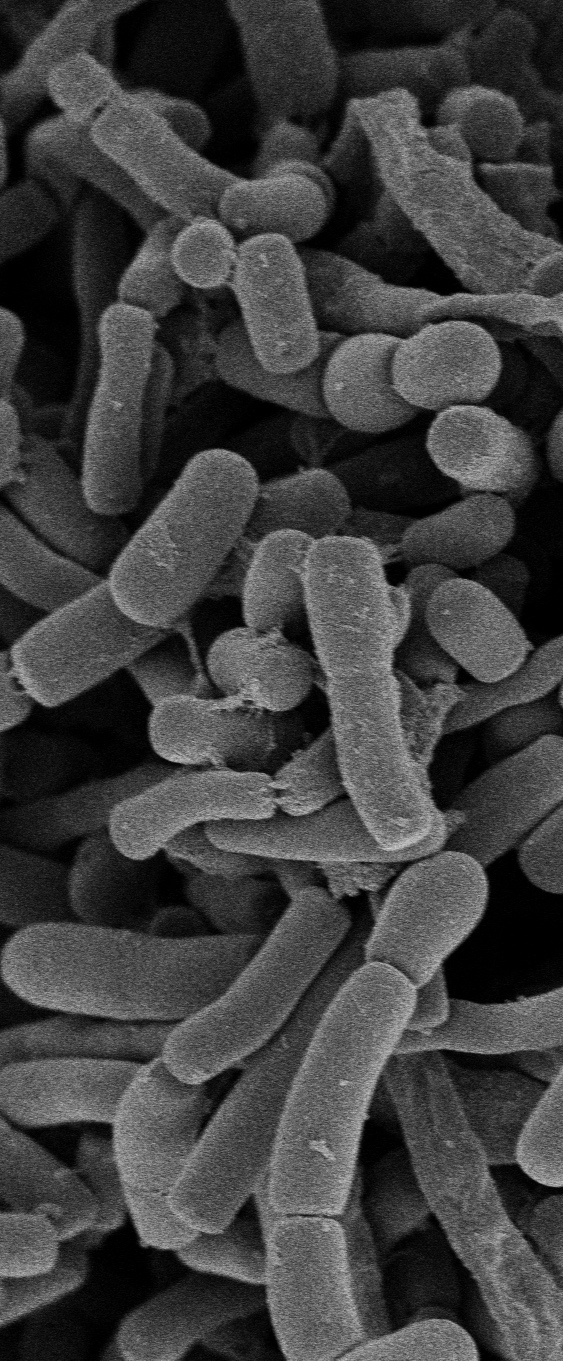


A2

b


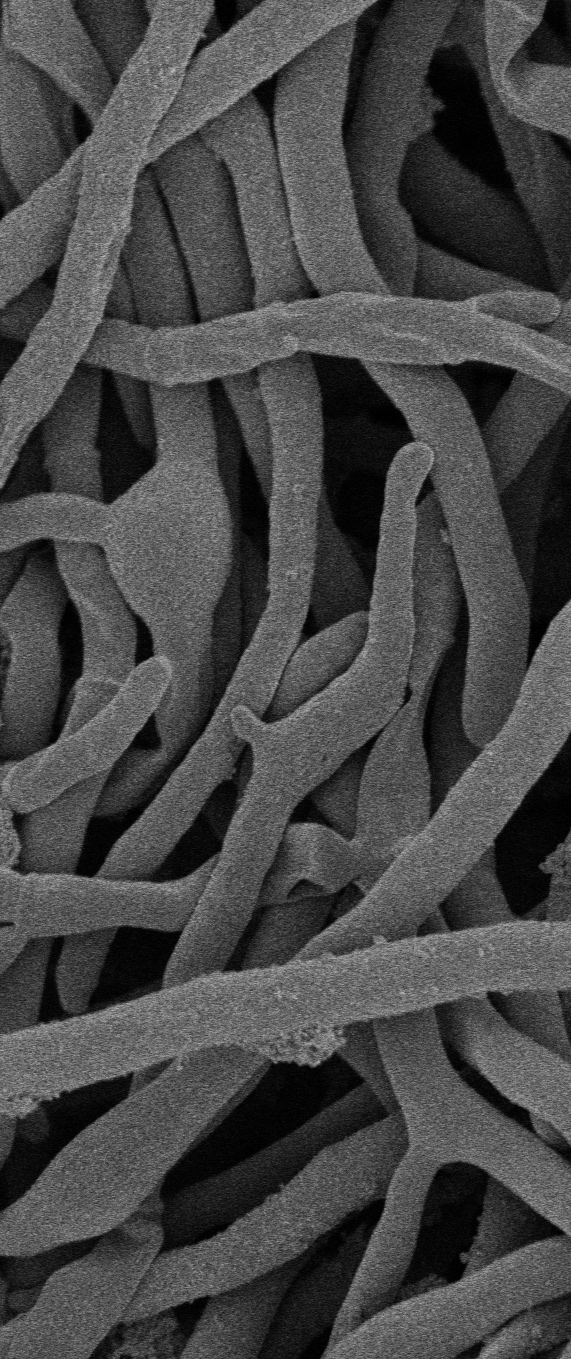


A1


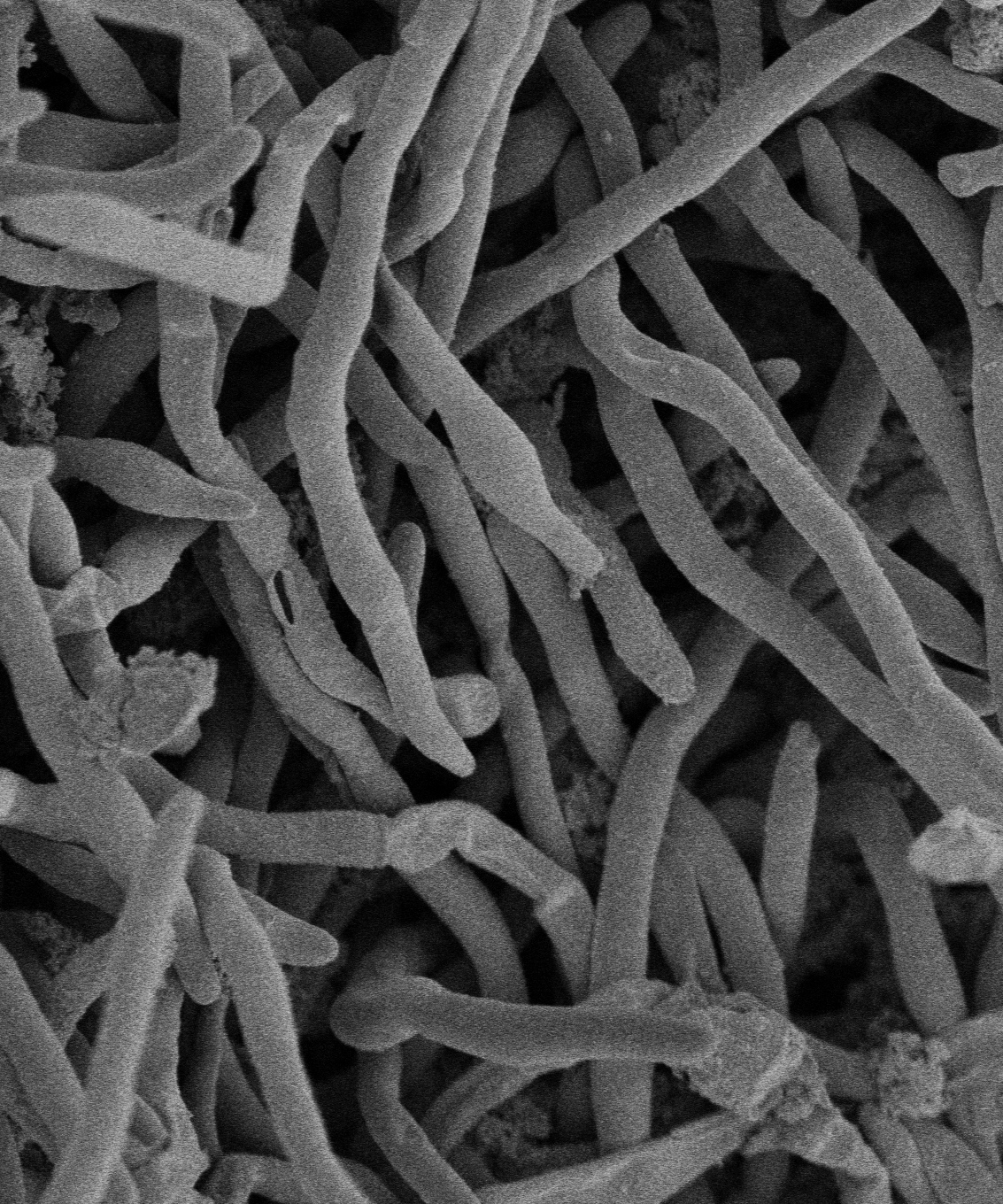


A1+A2


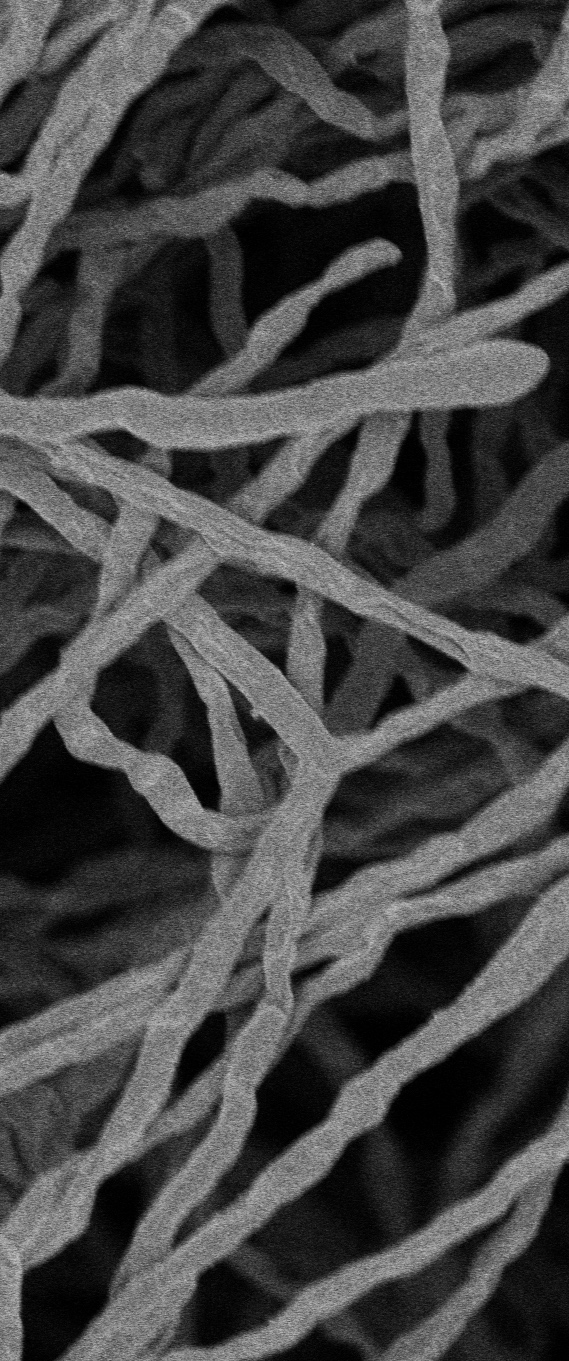


A2

**Supplemental figure 3**

**Scanning electron microscope image of co-isolated filamentous or cocci-shaped actinomycetes and other bacteria in co-culture.** (a) Left image: monoculture of HEK332A1. Middle image: co-culture of HEK332A1/A2. Right image: monoculture of HEK332A2. (b) Left image: monoculture of HEK423A1. Middle image: co-culture of HEK423A1/A2. Right image: monoculture of HEK423A2. (Scale bar = 2 µm)


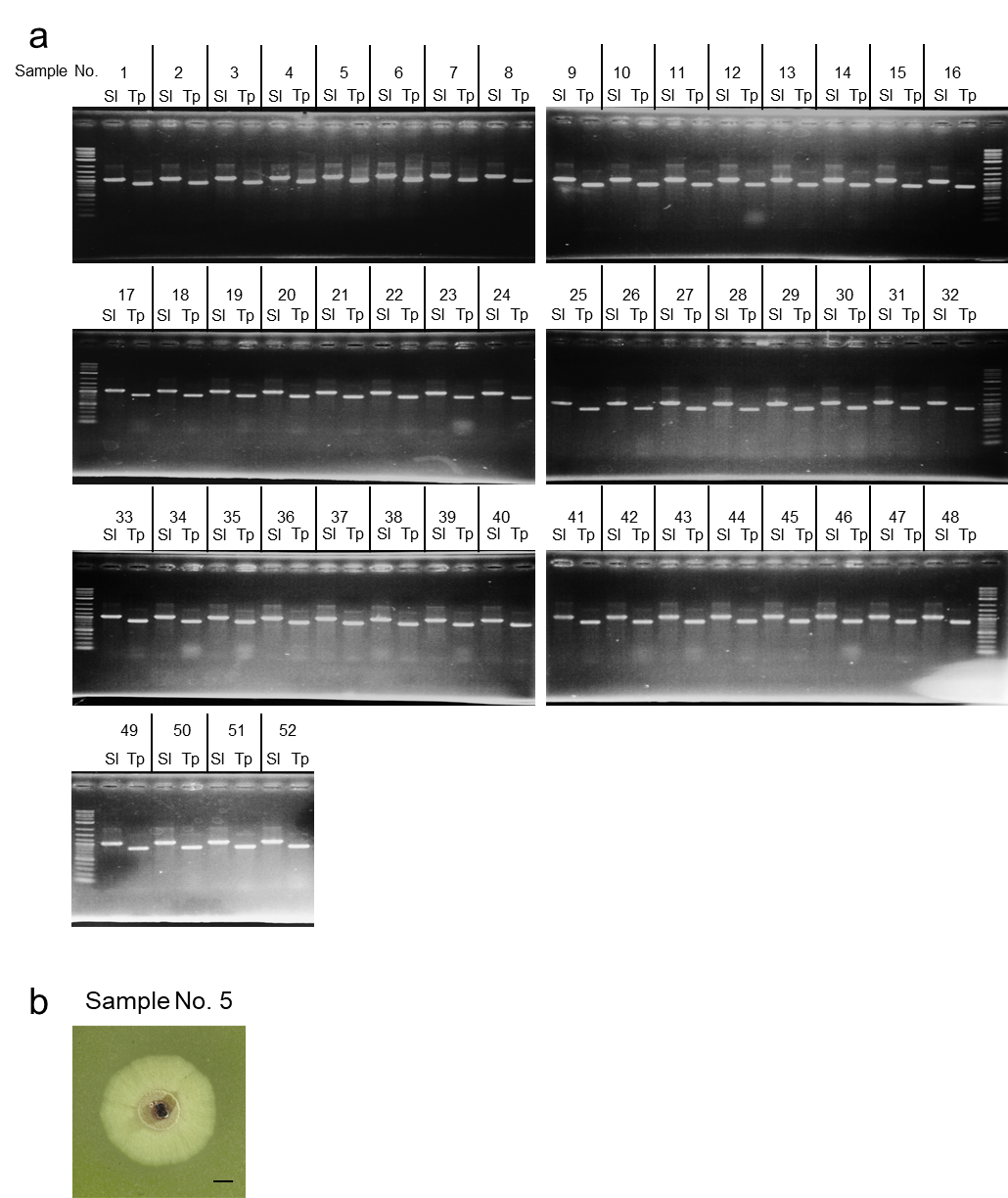
**Supplemental figure 4**

**Analysis of the colonies obtained by co-isolation experiments from combined-culture of *Streptomyces lividans* TK23 and *Tsukamurella pulmonis* TP-B0596.** (a) Agarose gel electrophoresis of selective colony PCR products. Fifty-two colonies were tested using primers to amplify specific regions of the 16S rRNA gene for *S. lividans* TK23 (Sl) and *T. pulmonis* TP-B0596 (Tp). (b) Example of stereomicroscope image of the colony containing both Sl and Tp. (Scale bar: 1 mm)


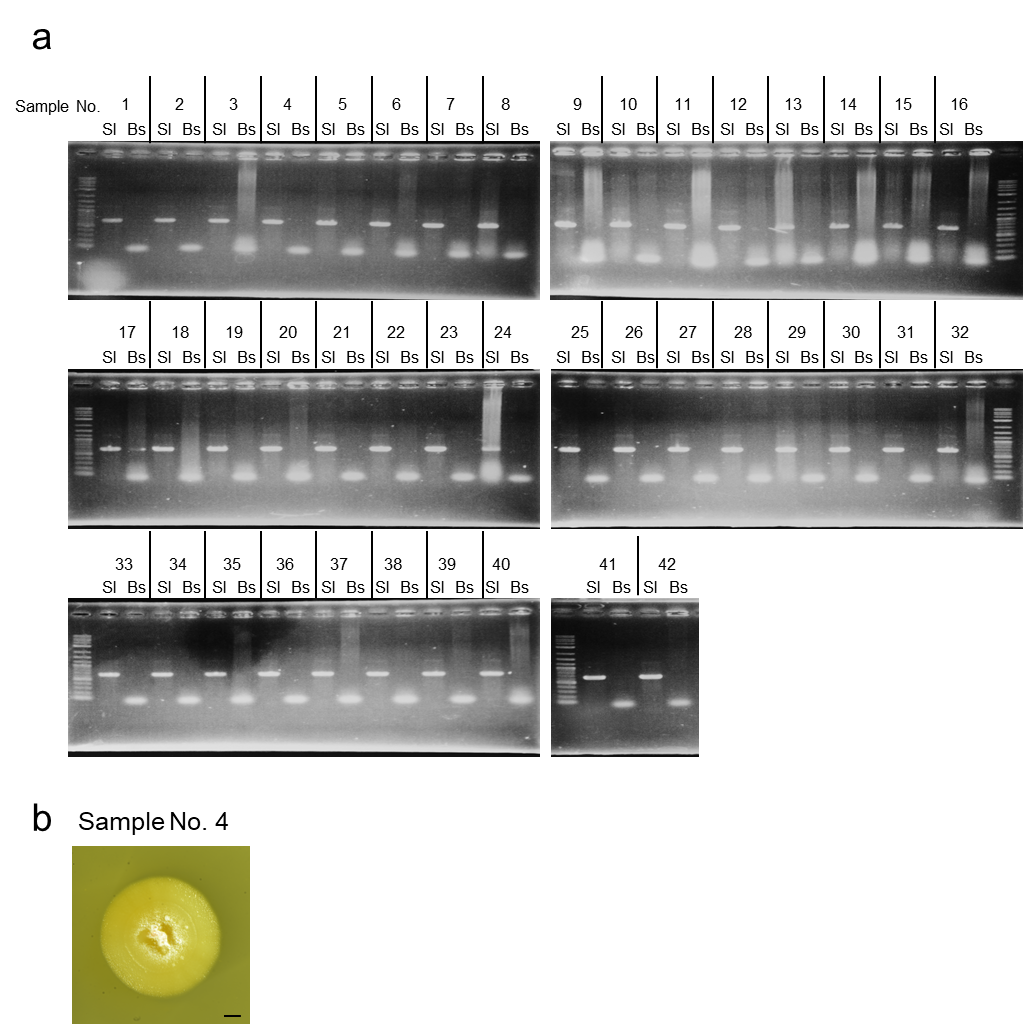
**Supplemental figure 5**

**Analysis of the colonies obtained by co-isolation experiments from co-culture of *Streptomyces lividans* TK23 and *Bacillus subtilis* 168.** (a) Agarose gel electrophoresis of selective colony PCR products. Forty-two colonies were tested using primers to amplify specific regions of the 16S rRNA gene for *S. lividans* TK23 (Sl) and *B. subtilis* 168 (Bs). (b) Example of stereomicroscope image of the colony containing both Sl and Bs. (Scale bar: 1 mm)


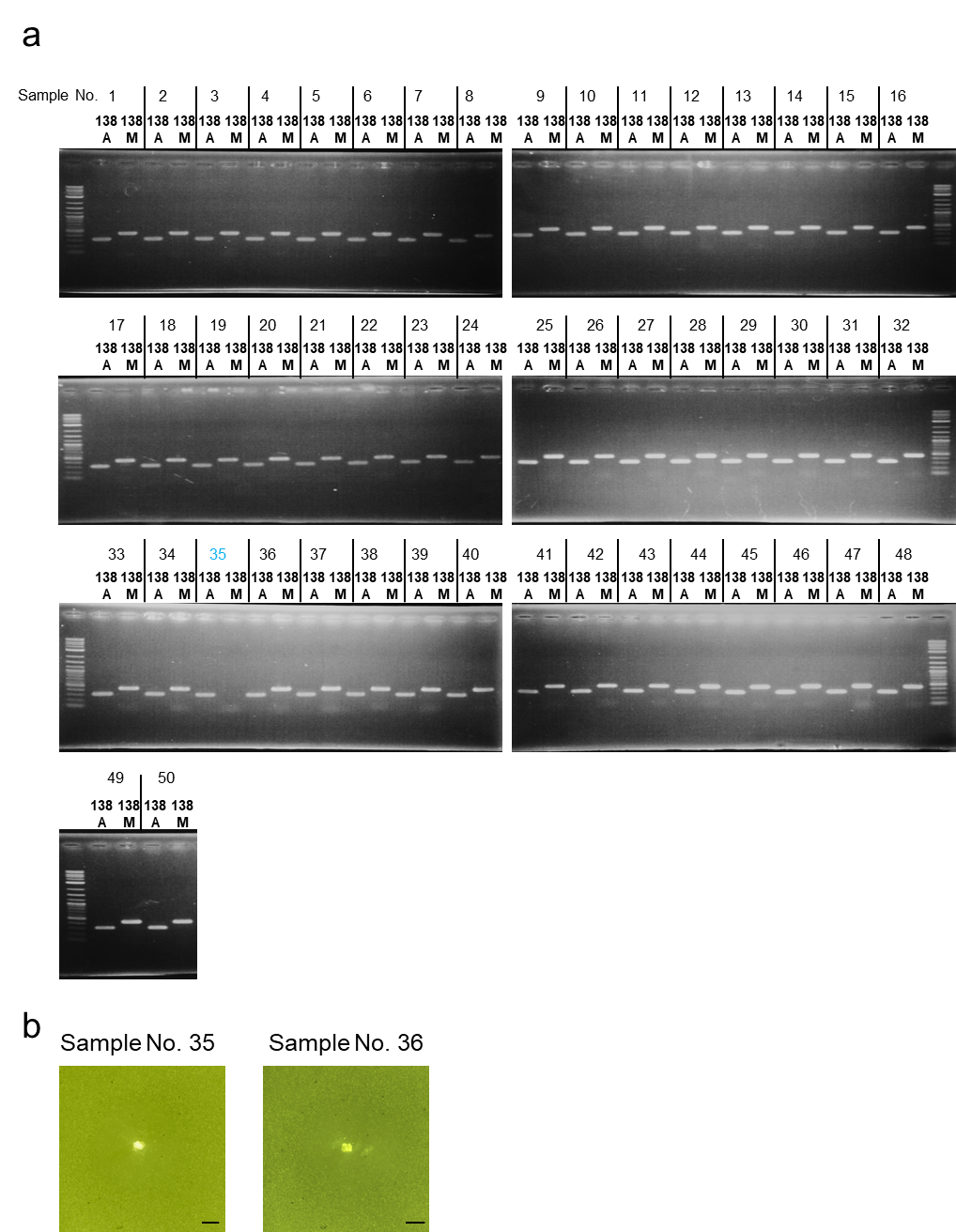
**Supplemental figure 6**

**Analysis of the colonies obtained by co-isolation experiments from combined culture of *Streptomyces* sp. HEK138A and *Mycobacterium* sp. HEK138M.** (a) Agarose gel electrophoresis of selective colony PCR products. Fifty colonies were tested using primers to amplify specific regions of the 16S rRNA gene for *Streptomyces* sp. HEK138A (138A) and *Mycobacterium* sp. HEK138M (138M). (b) Example of stereomicroscope image of the colony containing both 138A and 138M (No. 36), and stereomicroscope image of the colony containing 138A (No. 35). (Scale bar: 1 mm)


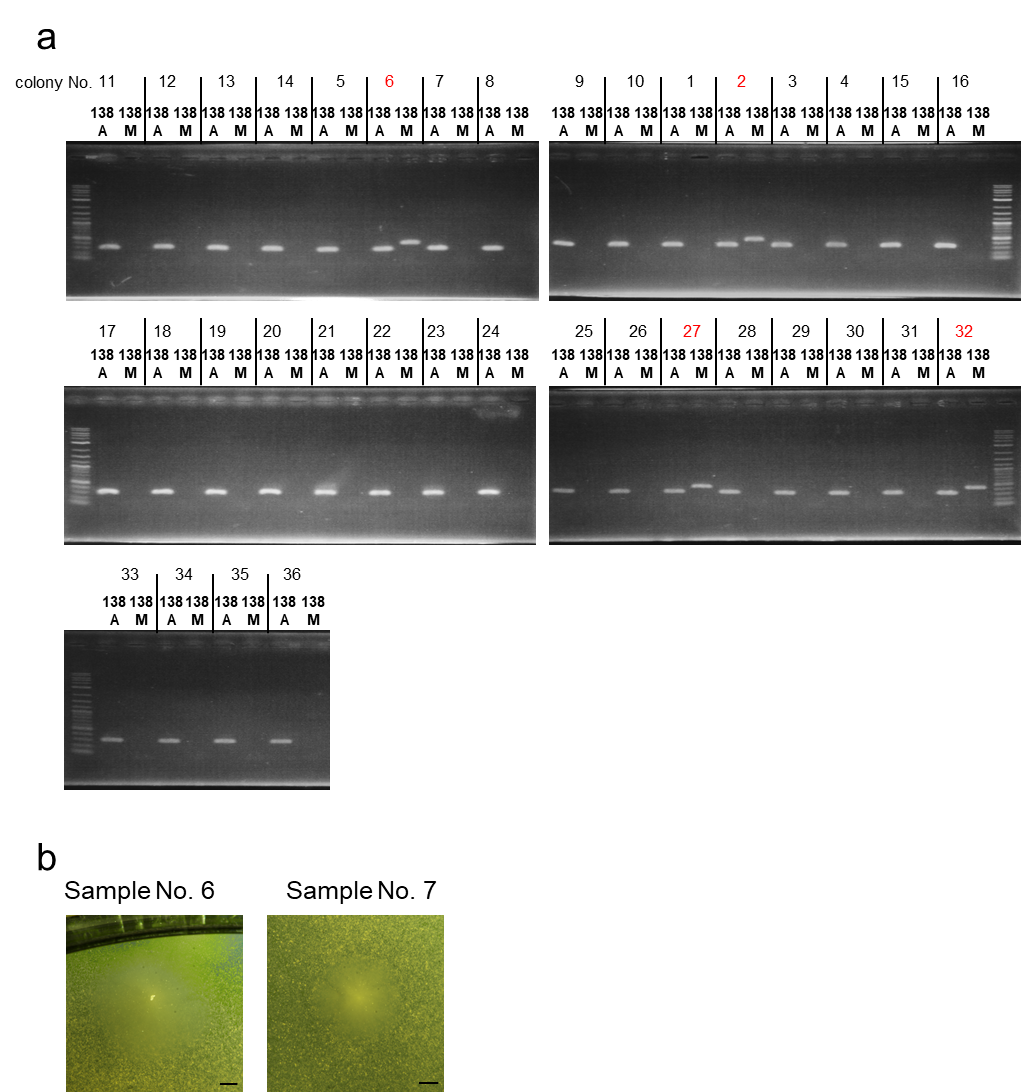
**Supplemental figure 7**

**Analysis of the colonies obtained by co-isolation experiments from combined-culture of *Streptomyces* sp. HEK138A and *Mycobacterium* sp. HEK138M from soil.** (a) Agarose gel electrophoresis of selective colony PCR products. Thirty-six colonies were tested using primers to amplify specific regions of the 16S rRNA gene for *Streptomyces* sp. HEK138A (138A) and *Mycobacterium* sp. HEK138M (138M). (b) Example of stereomicroscope image of the colony containing both 138A and 138M (No. 6), and stereomicroscope image of the colony containing 138A (No. 7). (Scale bar: 1 mm)


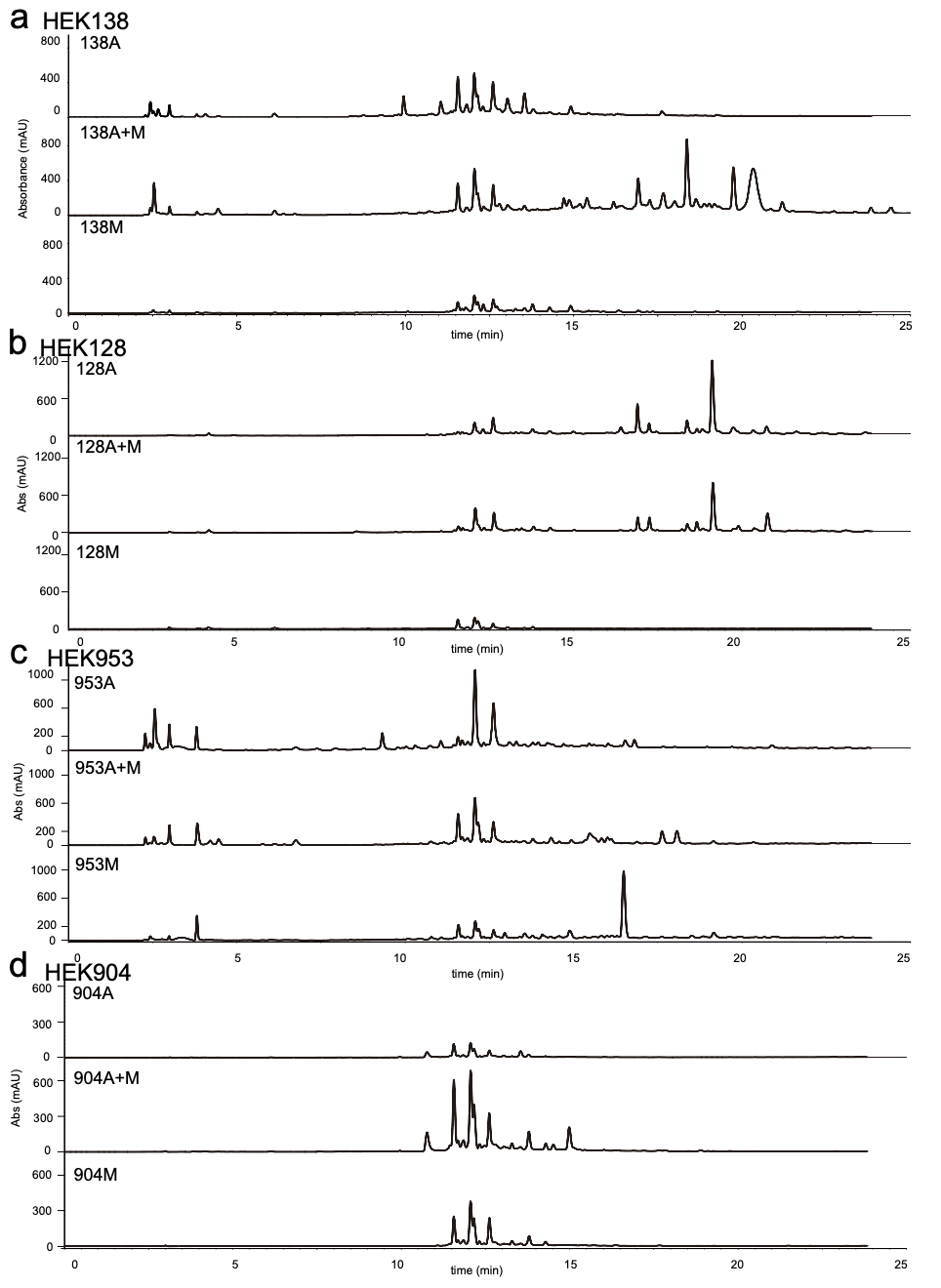
**Supplemental Figure 8**

High pressure liquid chromatography traces identical to the main manuscript Fig. 4 with different scale showing the whole chromatograms.


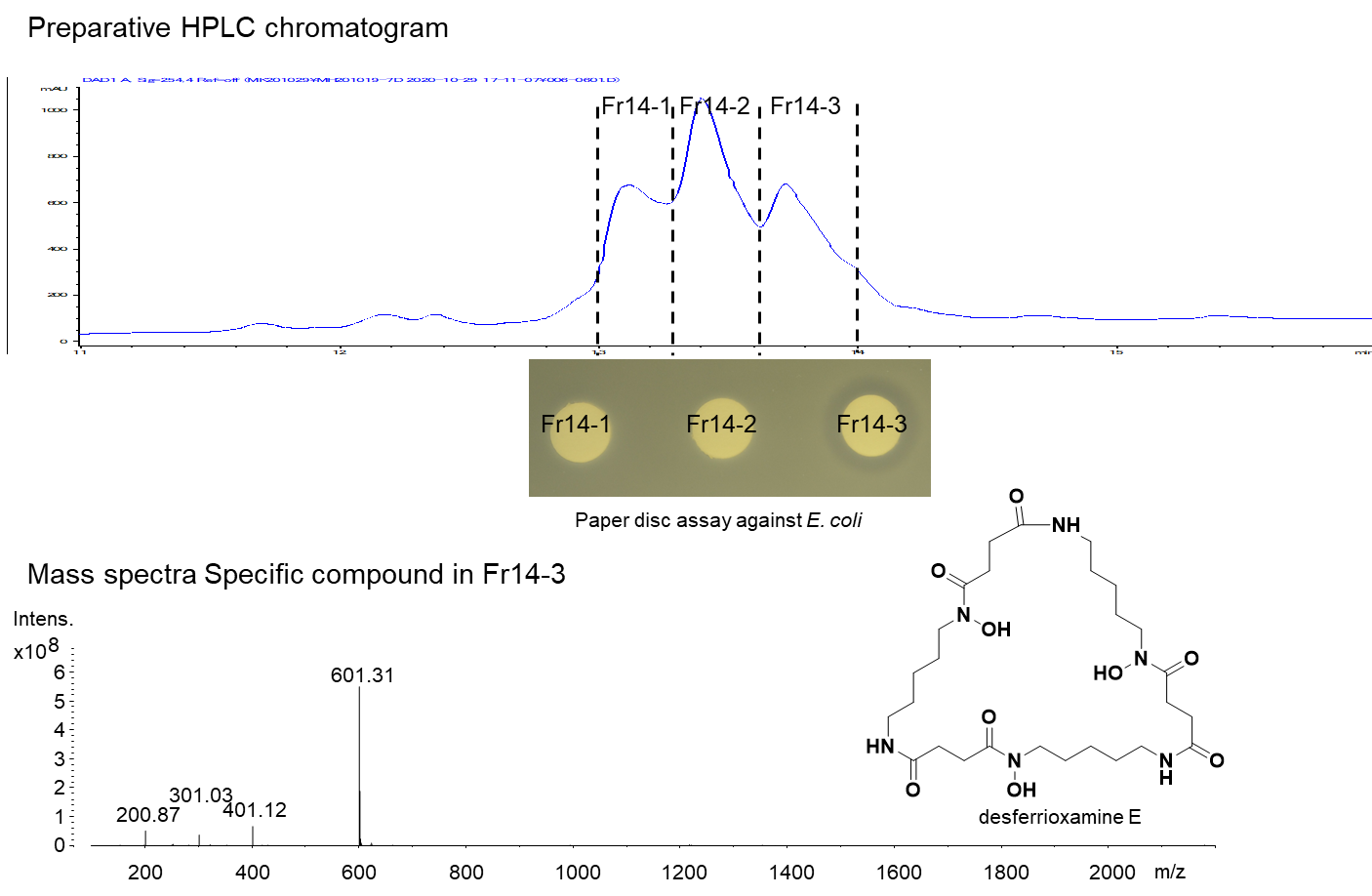
**Supplemental Figure 9**

**Identification of active compound from HEK138A and M.** HEK138A and 138M were precultured in 10 ml of V-22 medium in a test tube at 30°C, 180 rpm for 2 days. Then 1 ml of HEK138A and HEK138M were inoculated into 100 ml of A-3M medium in a K-1 flask at 30°C, 200 rpm for 5 days. A total of 0.7 L of the culture broth was extracted with an equal volume of BuOH to give 1.6 g of crude extract. The crude extract was dissolved in 20 ml of DMSO, and the active fraction was collected by using preparative high pressure liquid chromatography (HPLC; Agilent) equipped with a Cosmosil 5C18-AR-II column (10.0 i.d. ×250 mm). The mobile phase was 5% acetonitrile for the first 3 min, then increased to 95% by linear gradient from 3 to 27 min, and then maintained at 95% until 30 min. Flow rate was 3.0 ml/min, and the column temperature was 40°C. The sample (100 µl) was applied and the peaks eluted at 13.0–14.0 min showed growth inhibition against *Escherichia coli*. To further identify the active compound, preparative HPLC was performed 26 times and the peaks eluted at 13.0–14.0 min were collected. The collected fraction was dried in vacuo, and dissolved in 1.3 ml of DMSO. The sample (100 µl) was applied again to the preparative HPLC and the peak eluted at 13.8–14.0 min was collected twice and combined. The collected fraction was dried in vacuo, and the sample was dissolved in 100 μl of DMSO. The sample (20 µl) was used for bioassay. For mass spectrometry analysis, the sample was diluted 10-fold and 5 µl was applied for LC-ESI-MS (amaZon SL, Bruker). The HPLC system was equipped by Cosmosil 5C18-AR-II column (2.0 i.d. × 150 mm, Nacalai Tesque). Acetonitrile and H_2_O (MilliQ) containing 0.1% formic acid was used as the mobile phase. Acetonitrile was maintained at 5% for the first 0.5 min, then increased to 95% by linear gradient from 0.5 to 13.5 min, and then maintained at 95% until 15 min. The flow was 0.45 ml/min and the column temperature was 40°C. The active compound was determined to be desferrioxamine E (nocardamine) based on the MS/MS fragmentation pattern.^20^ Nocardamine is known to show weak growth inhibition against *E. coli*.^21^

**Supplemental table 4.** Sources and methods for the initial isolation from the Hegura Island environmental samples of tested bacteria used in this study.

| strain number |  | isolation point | isolation source | isolation methods | isolation medium |
| --- | --- | --- | --- | --- | --- |
| 128 | A | 20 | succulent plant growing root soil | H_2_O dilution | Humic acid |
|  | M |  |  |  |  |
| 138 | A | 21 | bamboo grass growing root soil | H_2_O dilution | ISP4 |
|  | M |  |  |  |  |
| 282 | A1 |  |  |  |  |
|  | A2 |  |  |  |  |
|  | B |  |  |  |  |
| 314 | A | 68 | Machilus thunbergii growing root soil | H_2_O dilution | Humic acid |
|  | B |  |  |  |  |
| 332 | A1 | 31 | soil | phenol treatment | ISP4 |
|  | A2 |  |  |  |  |
| 423 | A1 | 1 | soil | H_2_O dilution | ISP4 |
|  | A2 |  |  |  |  |
| 652 | A1 | 18 | Lichenes (ocher) growing soil | H_2_O dilution | ISP4 |
|  | A2 |  |  |  |  |
|  | B |  |  |  |  |
| 654 | A1 | 18 | Lichenes (ocher) growing soil | H_2_O dilution | ISP4 |
|  | A2 |  |  |  |  |
| 830 | A | 6 | soil | phenol treatment | ISP4 |
|  | B |  |  |  |  |
| 904 | A | 40 | soil | phenol treatment | ISP4 |
|  | M |  |  |  |  |
| 953 | A | 52 | seaweed | H_2_O dilution | Humic acid |
|  | M |  |  |  |  |

**Supplemental table 5.** Feature of collected environmental samples from the Hegura Island.

| isolation point | sample feature | isolation point | sample feature |
| --- | --- | --- | --- |
| 1 | soil | 37 | shell on rock |
| 2 | soil | 38 | Lichenes (green) growing soil |
| 3 | soil | 39 | soil |
| 4 | soil | 40 | soil |
| 5 | soil | 41 | seasand |
| 6 | soil | 42 | dead tree |
| 7 | soil | 43 | ivy growing soil |
| 8 | soil | 44 | Withered coral |
| 9 | soil | 45 | soil |
| 10 | soil | 46 | soil |
| 11 | soil | 47 | soil |
| 12 | soil | 48 | soil |
| 13 | soil | 49 | soil |
| 14 | dead tree (Angelica japonica) | 50 | soil |
| 15 | soil | 51 | driy Lichenes |
| 16 | soil | 52 | seaweed |
| 17 | imperatae rhizoma | 53 | succulent plant growing root soil |
| 18 | Lichenes (ocher) growing soil | 54 | succulent plant growing root soil |
| 19 | pond sediment (Ryujin-pond) | 55 | soil |
| 20 | succulent plant growing root soil | 56 | soil |
| 21 | bamboo grass growing root soil | 57 | puddle sediment |
| 22 | plantain growing root soil | 58 | soil |
| 23 | grass (unknown) growing root soil | 59 | soil |
| 24 | soil | 60 | dead crab |
| 25 | soil (kannondo stone wall) | 61 | soil |
| 26 | soil (kannondo stone wall) | 62 | bamboo grass growing root soil |
| 27 | soil (kannondo stone wall) | 63 | ivy growing soil |
| 28 | soil | 64 | Commelina communis growing root soil |
| 29 | soil | 65 | pine growing root soil |
| 30 | bamboo grass growing root soil | 66 | Japanese cherry growing root soil |
| 31 | soil | 67 | soil |
| 32 | pine growing root soil | 68 | Machilus thunbergii growing root soil |
| 33 | pteridophyte growing root soil | 69 | soil |
| 34 | soil | 70 | plant growing root soil |
| 35 | seaweed | 71 | soil |
| 36 | seasand sediment |  |  |


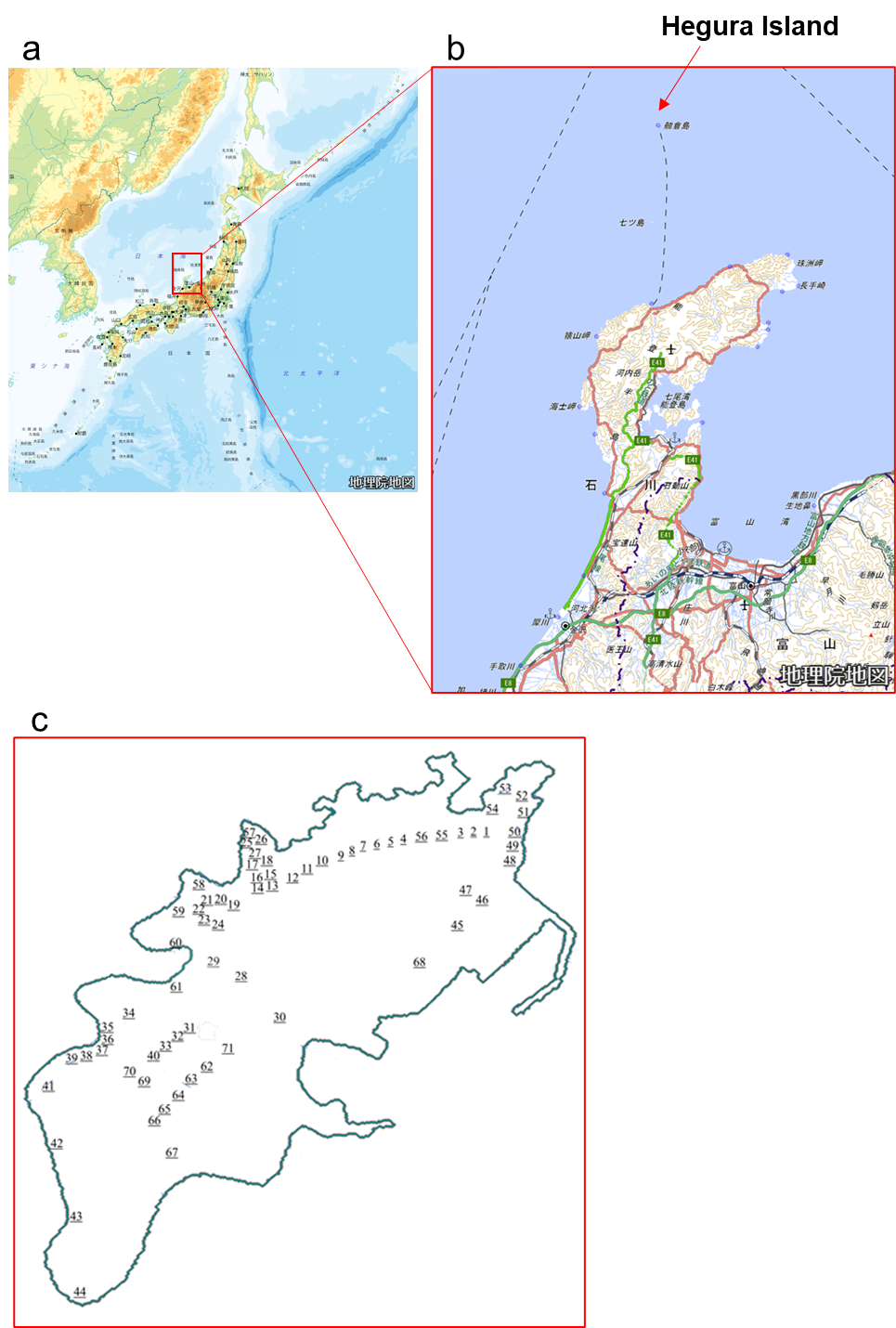


Supplemental figure 10

Sampling location and sampling points. (a) Location of Hegura Island in the Sea of Japan marked by framed box. (b) Enlarged view of Ishikawa, Japan and the location of Hegura Island pointed by arrow. Source of (a) and (b): Geospatial Information Authority of Japan website (https://maps.gsi.go.jp/). Maps were modified by a framed box and an arrow. The map (c) is a handwriting.

**References**

1. Hoshino, S.; Ozeki, M.; Wong, C. P.; Zhang, H.; Hayashi, F.; Awakawa, T.; Morita, H.; Onaka, H.; Abe, I., Mirilactams C-E, Novel Polycyclic Macrolactams Isolated from Combined-Culture of Actinosynnema mirum NBRC 14064 and Mycolic Acid-Containing Bacterium. *Chem Pharm Bull (Tokyo)* **2018,** *66* (6), 660-667.

2. Pan, C. Q.; Kuranaga, T.; Cao, X.; Suzuki, T.; Dohmae, N.; Shinzato, N.; Onaka, H.; Kakeya, H., Amycolapeptins A and B, Cyclic Nonadepsipeptides Produced by Combined-culture of Amycolatopsis sp. and Tsukamurella pulmonis. *J Org Chem* **2021,** *86* (2), 1843-1849.

3. Hoshino, S.; Ozeki, M.; Awakawa, T.; Morita, H.; Onaka, H.; Abe, I., Catenulobactins A and B, Heterocyclic Peptides from Culturing Catenuloplanes sp. with a Mycolic Acid-Containing Bacterium. *J Nat Prod* **2018,** *81* (9), 2106-2110.

4. Hoshino, S.; Okada, M.; Awakawa, T.; Asamizu, S.; Onaka, H.; Abe, I., Mycolic Acid Containing Bacterium Stimulates Tandem Cyclization of Polyene Macrolactam in a Lake Sediment Derived Rare Actinomycete. *Org Lett* **2017,** *19* (18), 4992-4995.

5. Jiang, Y. L.; Lu, S.; Hirai, G.; Kato, T.; Onaka, H.; Kakeya, H., Enhancement of saccharothriolide production and discovery of a new metabolite, saccharothriolide C-2, by combined-culture of Saccharothrix sp. and Tsukamurella pulmonis. *Tetrahedron Lett* **2019,** *60* (15), 1072-1074.

6. Oku, N.; Takemura, S.; Onaka, H.; Igarashi, Y., NMR characterization of streptogramin B and L-156,587, a non-synergistic pair of the streptogramin family antibiotic complexes produced inductively by a combined culture of Streptomyces albogriseolus and Tsukamurella pulmonis. *Magn Reson Chem* **2021**.

7. Hoshino, S.; Zhang, L.; Awakawa, T.; Wakimoto, T.; Onaka, H.; Abe, I., Arcyriaflavin E, a new cytotoxic indolocarbazole alkaloid isolated by combined-culture of mycolic acid-containing bacteria and Streptomyces cinnamoneus NBRC 13823. *J Antibiot (Tokyo)* **2015,** *68* (5), 342-4.

8. Hagihara, R.; Katsuyama, Y.; Sugai, Y.; Onaka, H.; Ohnishi, Y., Novel desferrioxamine derivatives synthesized using the secondary metabolism-specific nitrous acid biosynthetic pathway in Streptomyces davawensis. *J Antibiot (Tokyo)* **2018,** *71* (11), 911-919.

9. Igarashi, Y.; Kim, Y.; In, Y.; Ishida, T.; Kan, Y.; Fujita, T.; Iwashita, T.; Tabata, H.; Onaka, H.; Furumai, T., Alchivemycin A, a bioactive polycyclic polyketide with an unprecedented skeleton from Streptomyces sp. *Org Lett* **2010,** *12* (15), 3402-5.

10. Ozaki, T.; Sugiyama, R.; Shimomura, M.; Nishimura, S.; Asamizu, S.; Katsuyama, Y.; Kakeya, H.; Onaka, H., Identification of the common biosynthetic gene cluster for both antimicrobial streptoaminals and antifungal 5-alkyl-1,2,3,4-tetrahydroquinolines. *Org Biomol Chem* **2019,** *17* (9), 2370-2378.

11. Sugiyama, R.; Nishimura, S.; Ozaki, T.; Asamizu, S.; Onaka, H.; Kakeya, H., Discovery and Total Synthesis of Streptoaminals: Antimicrobial [5,5]-Spirohemiaminals from the Combined-Culture of Streptomyces nigrescens and Tsukamurella pulmonis. *Angew Chem Int Ed Engl* **2016,** *55* (35), 10278-82.

12. Sugiyama, R.; Nakatani, T.; Nishimura, S.; Takenaka, K.; Ozaki, T.; Asamizu, S.; Onaka, H.; Kakeya, H., Chemical Interactions of Cryptic Actinomycete Metabolite 5-Alkyl-1,2,3,4-tetrahydroquinolines through Aggregate Formation. *Angew Chem Int Edit* **2019,** *58* (38), 13486-13491.

13. Sugiyama, R.; Nishimura, S.; Ozaki, T.; Asamizu, S.; Onaka, H.; Kakeya, H., 5-Alkyl-1,2,3,4-tetrahydroquinolines, new membrane-interacting lipophilic metabolites produced by combined culture of Streptomyces nigrescens and Tsukamurella pulmonis. *Org Lett* **2015,** *17* (8), 1918-21.

14. Hoshino, S.; Wakimoto, T.; Onaka, H.; Abe, I., Chojalactones A-C, cytotoxic butanolides isolated from Streptomyces sp. cultivated with mycolic acid containing bacterium. *Org Lett* **2015,** *17* (6), 1501-4.

15. Jiang, Y. L.; Matsumoto, T.; Kuranaga, T.; Lu, S.; Wang, W. C.; Onaka, H.; Kakeya, H., Longicatenamides A-D, Two Diastereomeric Pairs of Cyclic Hexapeptides Produced by Combined-culture of Streptomyces sp. KUSC_F05 and Tsukamurella pulmonis TP-B0596. *J Antibiot* **2021**.

16. Hoshino, S.; Okada, M.; Wakimoto, T.; Zhang, H.; Hayashi, F.; Onaka, H.; Abe, I., Niizalactams A-C, Multicyclic Macrolactams Isolated from Combined Culture of Streptomyces with Mycolic Acid-Containing Bacterium. *J Nat Prod* **2015,** *78* (12), 3011-7.

17. Hoshino, S.; Wong, C. P.; Ozeki, M.; Zhang, H.; Hayashi, F.; Awakawa, T.; Asamizu, S.; Onaka, H.; Abe, I., Umezawamides, new bioactive polycyclic tetramate macrolactams isolated from a combined-culture of Umezawaea sp. and mycolic acid-containing bacterium. *J Antibiot (Tokyo)* **2018,** *71* (7), 653-657.

18. Ozaki, T.; Kurokawa, Y.; Hayashi, S.; Oku, N.; Asamizu, S.; Igarashi, Y.; Onaka, H., Insights into the Biosynthesis of Dehydroalanines in Goadsporin. *Chembiochem* **2016,** *17* (3), 218-223.

19. Onaka, H.; Ozaki, T.; Mori, Y.; Izawa, M.; Hayashi, S.; Asamizu, S., Mycolic acid-containing bacteria activate heterologous secondary metabolite expression in Streptomyces lividans. *J Antibiot (Tokyo)* **2015,** *68* (9), 594-7.

20. Traxler, M. F.; Watrous, J. D.; Alexandrov, T.; Dorrestein, P. C.; Kolter, R., Interspecies interactions stimulate diversification of the Streptomyces coelicolor secreted metabolome. *mBio* **2013,** *4* (4).

21. Anke, H., Metabolic Products of Microorganisms .163. Desferritriacetylfusigen, an Antibiotic from Aspergillus-Deflectus. *J Antibiot* **1977,** *30* (2), 125-128.
